# A recessive *PRDM13* mutation results in congenital hypogonadotropic hypogonadism and cerebellar hypoplasia

**DOI:** 10.1172/JCI141587

**Published:** 2021-12-15

**Authors:** Danielle E. Whittaker, Roberto Oleari, Louise C. Gregory, Polona Le Quesne-Stabej, Hywel J. Williams, GOSgene, John G. Torpiano, Nancy Formosa, Mario J. Cachia, Daniel Field, Antonella Lettieri, Louise A. Ocaka, Alyssa J.J. Paganoni, Sakina H. Rajabali, Kimberley L.H. Riegman, Lisa B. De Martini, Taro Chaya, Iain C.A.F. Robinson, Takahisa Furukawa, Anna Cariboni, M. Albert Basson, Mehul T. Dattani

**Affiliations:** 1 Centre for Craniofacial and Regenerative Biology, King’s College London, London, United Kingdom.; 2 Department of Comparative Biomedical Sciences, Royal Veterinary College, London, United Kingdom.; 3 Department of Pharmacological and Biomolecular Sciences, University of Milan, Milan, Italy.; 4 Section of Molecular Basis of Rare Disease, Genetics and Genomic Medicine Research and Teaching Department, UCL Great Ormond Street Institute of Child Health, London, United Kingdom.; 5 GOSgene is detailed in Supplemental Acknowledgments.; 6 Department of Paediatrics and; 7 Adult Endocrinology Service, Mater Dei Hospital, Msida, Malta.; 8 Laboratory for Molecular and Developmental Biology, Institute for Protein Research, Osaka University, Osaka, Japan.; 9 The Francis Crick Institute, London, United Kingdom.; 10 MRC Centre for Neurodevelopmental Disorders, King’s College London, London, United Kingdom.

**Keywords:** Development, Endocrinology, Genetic diseases, Neurodevelopment, Neuroendocrine regulation

## Abstract

The positive regulatory (PR) domain containing 13 (PRDM13) putative chromatin modifier and transcriptional regulator functions downstream of the transcription factor PTF1A, which controls GABAergic fate in the spinal cord and neurogenesis in the hypothalamus. Here, we report a recessive syndrome associated with *PRDM13* mutation. Patients exhibited intellectual disability, ataxia with cerebellar hypoplasia, scoliosis, and delayed puberty with congenital hypogonadotropic hypogonadism (CHH). Expression studies revealed *Prdm13/PRDM13* transcripts in the developing hypothalamus and cerebellum in mouse and human. An analysis of hypothalamus and cerebellum development in mice homozygous for a *Prdm13* mutant allele revealed a significant reduction in the number of Kisspeptin (Kiss1) neurons in the hypothalamus and PAX2^+^ progenitors emerging from the cerebellar ventricular zone. The latter was accompanied by ectopic expression of the glutamatergic lineage marker TLX3. *Prdm13*-deficient mice displayed cerebellar hypoplasia and normal gonadal structure, but delayed pubertal onset. Together, these findings identify PRDM13 as a critical regulator of GABAergic cell fate in the cerebellum and of hypothalamic kisspeptin neuron development, providing a mechanistic explanation for the cooccurrence of CHH and cerebellar hypoplasia in this syndrome. To our knowledge, this is the first evidence linking disrupted PRDM13-mediated regulation of Kiss1 neurons to CHH in humans.

## Introduction

Congenital hypogonadotropic hypogonadism (CHH) is a rare genetic disorder caused by defective development or functioning of hypothalamic gonadotrophin-releasing hormone–secreting (GnRH-secreting) neurons, leading to deficiency of GnRH, the master hormone of the reproductive axis (1). During embryogenesis, GnRH neurons originating in the nasal placode migrate into the brain (2, 3) and innervate the median eminence to release GnRH. GnRH neurons receive excitatory and inhibitory inputs from other hypothalamic neurons, including Kisspeptin (Kiss1) neurons (4, 5), which mainly promote GnRH secretion, and gonadotropin-inhibitory hormone (GnIH) neurons (6), which instead suppress GnRH neuronal activity. The interaction of their signalling pathways is proposed to fine-tune GnRH neuronal activity and, consequently, pulsatile gonadotropin secretion from the pituitary gland (6).

CHH is clinically characterized by complete or partial absence of puberty and impaired or absent fertility. Additional clinical signs can be present in multisyndromic forms of CHH (7), including cerebellar atrophy and ataxia (8–15). Cerebellar atrophy in these conditions is thought to be caused by neurodegeneration. Thus far, developmental syndromes characterized by the cooccurrence of CHH and cerebellar hypoplasia have not been reported.

Early embryonic defects in cerebellar patterning have been linked to hypoplasia of the medial cerebellum and the vermis (16, 17), while alterations in neurogenesis tend to affect both the vermis and hemispheres (18–20). Cerebellar neurons derive from two germinal zones, the rhombic lip and the ventricular zone (VZ). Glutamatergic neurons are specified in the rhombic lip, while GABAergic neurons originate in the VZ. The basic helix-loop-helix transcription factors ATOH1 and PTF1A contribute to the spatial segregation of these germinal zones, respectively (18, 20–22). While ATOH1 deletion leads to failure of glutamatergic progenitors to expand (20), PTF1A deficiency results in the misspecification of GABAergic VZ progenitors and aberrant expression of glutamatergic fate markers (19, 23).

PR domain containing 13 (*PRDM13*) belongs to the PRDM family of transcriptional regulators, defined by a positive regulatory (PR) domain and a variable number of zinc finger domains (24, 25). PRDM factors modulate transcriptional activity by acting either as direct histone methyltransferases via catalytic activity of their PR domains (26, 27) or by recruiting other histone-modifying enzymes to chromatin (28–31). They are involved in many developmental processes that drive and maintain cell-state transitions or that modify the activity of signaling pathways (32). PRDM13 functions as an essential GABAergic cell-fate determinant in the spinal cord and the retina (33–35). In the spinal cord, PRDM13 functions downstream of PTF1A to promote GABAergic and suppress glutamatergic fate (35, 36).

PTF1A is a critical VZ specification factor during early cerebellum development, where it is both necessary and sufficient for the development of GABAergic Purkinje cells (PCs) and interneurons (18, 19). Recessive *PTF1A* mutations are associated with pancreatic and cerebellar agenesis/hypoplasia (37). Furthermore, PTF1A was recently found to be required for the development of the hypothalamus in the mouse, where its forebrain-specific deletion leads to disruption of the Kiss1 neuronal system, hypogonadism, and altered sexual behaviors (38). These findings suggest the possibility that *PTF1A* or *PRDM13* mutations may be responsible for some congenital disorders characterized by both cerebellar hypoplasia and CHH.

Here, we report a recessive syndrome associated with a *PRDM13* mutation in unrelated patients exhibiting intellectual disability, ataxia with cerebellar hypoplasia, scoliosis, and delayed puberty with CHH. By combining exome sequencing on human patients with phenotypic analysis of *Prdm13*-deficient mice, we identified critical neurodevelopmental functions for PRDM13 that underlie these reproductive and cerebellar phenotypes. Our results are consistent with a conserved PTF1A/PRDM13 regulatory axis controlling cell-fate specification in several neurogenic niches in the developing brain, including the hypothalamus and cerebellum.

## Results

### A recessive syndrome with PRDM13 mutation.

**Figure 1:**
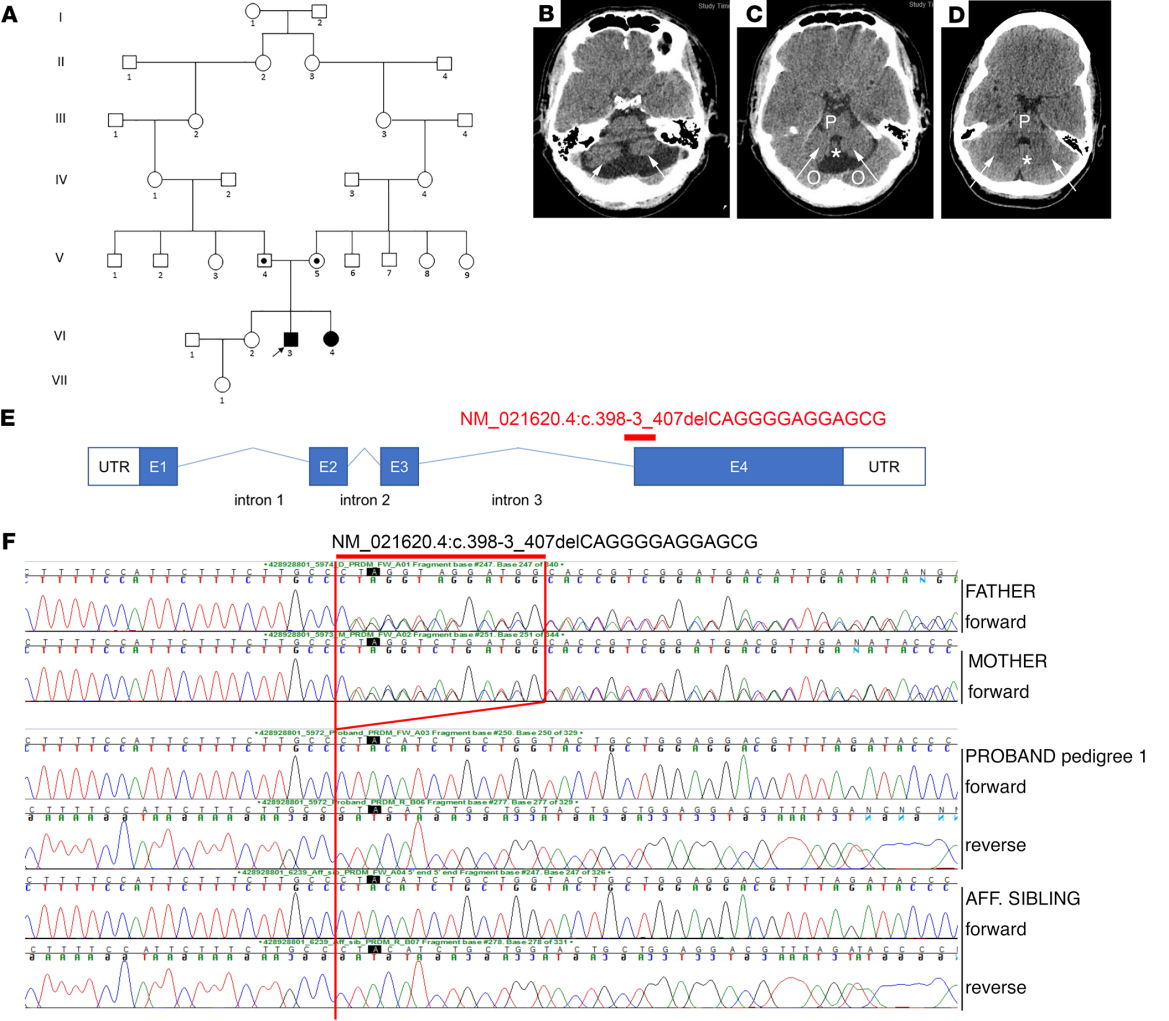
Exome sequencing identifies a *PRDM13* mutation in 3 patients from 2 Maltese pedigrees. (**A**) Pedigree 1 with 1 affected male (VI.3) (patient 1) and 1 affected female (VI.4) (patient 2) with a syndrome associated with HH and cerebellar hypoplasia carrying a homozygous *PRDM13* mutation. Circles denote females; squares denote males; black square denotes affected male, and black circle denotes affected female; a dot in the middle of a shape indicates a heterozygous carrier; arrow indicates the proband. (**B**–**D**) (**B** and **C**) Axial slices on CT scan of the brain showing cerebellar hypoplasia in patient 1 compared with a normal CT scan from an unrelated individual (**D**). Arrows in **B** and **C** demonstrate hypoplastic cerebellar lobes as compared with those in the control scan (**D**). The pons (P) is hypoplastic compared with the scan in **D**, as is the cerebellar vermis (asterisk). Partial voluming from the occipital lobes above the tentorium is seen (O). (**D**) CT scan showing normal cerebellar lobes (arrows), a normal cerebellar vermis (asterisk), and a normal pons (P). (**E**) Diagram of *PRDM13* transcript (NM_021620.4) showing the deletion found in patients at the intron 3/exon 4 border, which is predicted to affect splicing and to form a truncated PRDM13 protein. (**F**) Electropherograms of patients 1 and 2 and their unaffected parents (pedigree 1) showing the c.398-3_407delCAGGGGAGGAGCG deletion homozygous in the 2 patients and heterozygous in the healthy parents. Aff, affected.

We identified 3 patients (2 male, 1 female) from 2 unrelated families in Malta presenting with similar clinical features. Patients 1 and 2 (male and female, respectively) were born to a consanguineous union and patient 3 (male) to nonconsanguineous parents (Figure 1A). Patients presented with delayed motor development, ataxia, scoliosis, intellectual disability, and delayed sexual development (Supplemental Table 1; supplemental material available online with this article; https://doi.org/10.1172/JCI141587DS1). All patients required corrective surgery for scoliosis and presented with either generalized hypotonia with hyporeflexia (patients 1 and 2) or hypertonia with hyperreflexia (patient 3). Further details outlining the neurological findings are shown in Supplemental Tables 1 and 2. Patients 1 and 3 also had cerebellar hypoplasia on neuroimaging (Figure 1, B–D).

CHH was diagnosed based on a combination of clinical and biochemical data. Patient 1 had bilateral undescended testes and underwent bilateral orchidopexies, which was repeated on the left at 6 years of age. CT scans of the brain revealed hypoplasia of the cerebellar hemispheres and vermis (Figure 1B). Progression of scoliosis necessitated a 2-stage surgical fixation of the spine at the age of 10 years. He was referred to the Paediatric Endocrine Clinic, Mater Dei Hospital, at 14.3 years with delayed puberty. Standard Tanner pubertal staging was conducted at this age and was G1 P1 A1 –/03 mL. A GnRH test revealed a peak luteinizing hormone (LH) of 2.3 IU/L, with a follicle-stimulating hormone (FSH) of 4.4 IU/L. A 3-day human chorionic gonadotrophin (hCG) test revealed no change in testosterone concentration after 3 hCG injections (peak testosterone of 2.2 nmol/L) and was therefore suboptimal and consistent with CHH. We have previously reported that a peak LH to GnRH stimulation of less than 2.8 IU/L, peak 3-day testosterone cut-off of less than 1.04 μg/L (3.6 nmol/L), and a peak 3-week testosterone cut-off of less than 2.75 μg/L (9.5 nmol/L) gave a sensitivity of 88% and a specificity of 100% for the diagnosis of CHH (39). Treatment was commenced with testosterone supplementation at the age of 14.5 years in order to allow development of secondary sexual characteristics.

Patient 2 was noted to have generalized hypotonia and hyporeflexia as well as delayed gross motor development. A progressive right-sided thoracolumbar scoliosis was first noted at the age of 2 years. All neurological investigations, including metabolic screen, electromyography (EMG), and brain MRI, were reported as normal. She was first seen in the Paediatric Endocrinology Clinic at age 11.3 years, with a neurological condition similar to that of her elder brother (patient 1). She had not entered spontaneous puberty by the age of 12.5 years, with low basal gonadotrophins (basal LH <0.1 U/L, FSH 0.8 IU/L) and with a peak LH of 2.6 IU/L and an FSH of 6.4 IU/L on GnRH testing. Few data are available for cut-off values for the diagnosis of CHH in females, but these gonadotrophin responses would be considered to be suboptimal in a 12.5-year-old girl with no signs of puberty, as would the undetectable basal LH concentration. In order to achieve development of secondary sexual characteristics, estrogen supplementation was commenced at the age of 13 years.

Patient 3 presented with global developmental delay, generalized hypertonia, and hyperreflexia at 3 months of age. A CT brain scan revealed cerebellar hypoplasia, and he had a broad-based gait. He needed corrective surgery for strabismus as well as spinal surgery for progressive scoliosis at 11 years of age, but became completely wheelchair dependent by age 12 years. He was referred to the Paediatric Endocrine Clinic at age 11.4 years with a micropenis. On pubertal staging at this age, his stretched penile length was 4 cm (less than P10) and both testes were impalpable. Basal gonadotrophins were low (LH <0.1 IU/L, FSH 0.4 IU/L). A GnRH test performed at the age of 13 years revealed a peak LH of 0.9 IU/L with an FSH of 3.1 IU/L. The peak testosterone was suboptimal at 2.3 nmol/L after a 3-day hCG test, with an excellent peak of 30.7 nmol/L after 3 weeks of HCG. The suboptimal LH together with suboptimal response to 3 days of HCG and the micropenis with undescended testes support a diagnosis of CHH. Following these tests, the left testis descended into the scrotum (2 mL volume), but the right testis remained impalpable. Treatment with low-dose intramuscular testosterone enantate was commenced at 13.5 years of age, and the dose increased gradually over the following 2 years. Bilateral orchidopexies were performed at 14 years of age. Over time, he experienced penile growth, but both testes remained 2 mL in volume.

### Whole-exome sequencing of patients 1 and 2 identified a 13 bp deletion in PRDM13.

The homozygous *PRDM13* deletion NC_000006.11:g. 100060906_ 100060918del (c.398-3_407delCAGGGGAGGAGCG), located at chr6:100060906 (GRCh37), spans the intron 3/exon 4 boundary (Figure 1E). This pathogenic variant is predicted to affect splicing, with premature truncation of PRDM13 resulting in the loss of all 4 zinc finger domains according to MutationTaster software (https://www.mutationtaster.org/) (40). This variant was confirmed by Sanger sequencing to be homozygous in patients 1 and 2 and heterozygous in the unaffected parents (Figure 1F). Given the phenotypic similarity of patient 3 to patients 1 and 2, we opted to perform Sanger sequencing of *PRDM13* in patient 3. This confirmed the presence of the same homozygous 13 bp deletion in this patient (Figure 1F). His unaffected parents were also heterozygous for the deletion. This mutation was not present in control databases, including the gnomAD browser (https://gnomad.broadinstitute.org/) (~123 K samples). To determine whether this mutation might be present in the Maltese population, 42 Maltese control individuals were screened for this *PRDM13* variant, one of whom was a heterozygous carrier. To delineate the region harboring the 13 bp deletion in more detail, we performed genome-wide microarray analysis of the 3 patients and an unrelated heterozygous Maltese carrier. This revealed that the 3 patients shared an identical region of homozygosity, spanning approximately 1.6 Mb, that encompassed the 13 bp deletion. The heterozygous carrier shared a smaller nested region of homozygosity of 0.2 Mb, also encompassing the 13 bp deletion (Supplemental Table 3).

### A homozygous Prdm13 mouse model for investigating nonlethal phenotypes.

Mice homozygous for *Prdm13*-null mutations are perinatal lethal (35), suggesting that the *PRDM13* mutations in these patients are most likely partial loss of function. The only homozygous *Prdm13* mutations thus far reported that do not cause perinatal lethality in mice are those with frameshift mutations in exon 1 or deletion of exons 2 and 3 (33, 35). We therefore generated homozygous *Prdm13* mutants carrying targeted deletion of exons 2 and 3, encoding the majority of the PR domain (Supplemental Figure 1, A and B). Quantitative reverse-transcriptase PCR (qRT-PCR) confirmed the absence of exon 2 and 3 transcripts and the presence of exon 4–containing transcripts in *Prdm13*^Δ^*ex2,3/*^Δ*ex2,3*^ mutant cerebella at E12.5 (Supplemental Figure 1C). Immunostaining of cerebellar tissue from these mice with antiserum raised against the C-terminal fragment of the protein (aa 685–754) confirmed the absence of this epitope. These *Prdm13*^Δ^*ex2,3/*^Δ*ex2,3*^ mutants, referred to from here on as *Prdm13–/–* mutants, survived to adulthood as previously reported (33). *Prdm13–/–* mice did not exhibit any signs suggestive of a neurodegenerative disease associated with aging (age range, 8–12 months, *n* = 9).

### Prdm13 loss does not affect the development of GnRH neurons.

**Figure 2:**
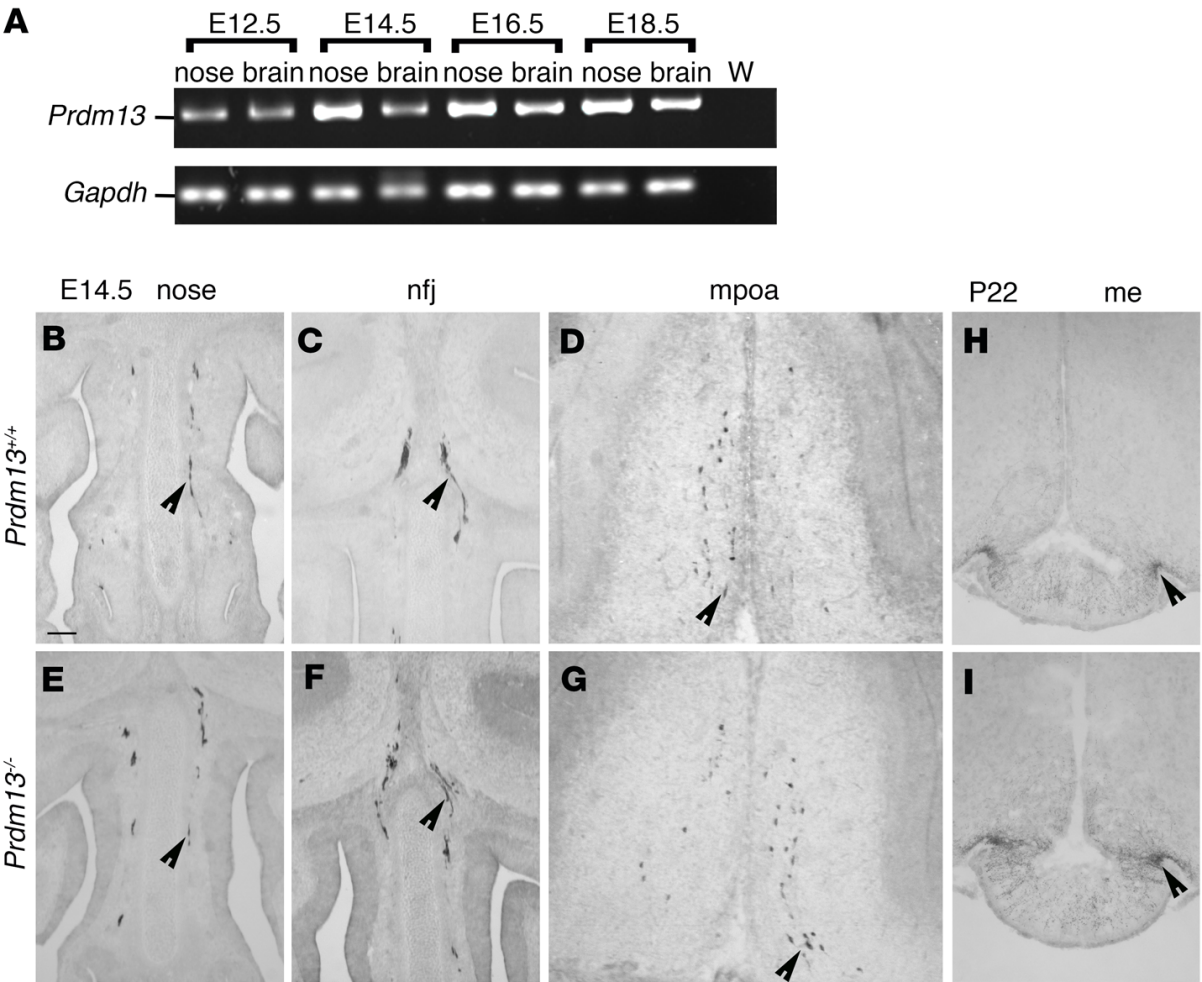
GnRH neuron specification and migration are not affected in *Prdm13* mutant mice. (**A**) RT-PCR analysis of *Prdm13* expression in mouse embryonic nose and brain tissues extracted from indicated embryonic stages of WT mouse embryos. *Gapdh* expression serves as positive control. W, water-only control, no cDNA. (**B**–**G**) Coronal sections of *Prdm13+/+* and *Prdm13–/–* E14.5 heads, immunolabeled for GnRH to reveal GnRH neurons in the nasal compartment (**B** and **E**), in the nfj (**C** and **F**), and in the mpoa (**D** and **G**). Black arrowheads indicate examples of neurons migrating in the nasal compartment (**B**–**E**), crossing the olfactory bulbs (**C** and **F**), and reaching the mpoa (**D** and **G**). (**H** and **I**) Coronal sections of *Prdm13+/+* and *Prdm13–/–* P22 brains immunolabeled for GnRH to reveal median eminence (me) innervation by GnRH neuron neurites, indicated by black arrowheads. Scale bar: 250 μm.

As patients carrying homozygous mutations of *PRDM13* display gonadotropin deficiency (Supplemental Table 1), we first analyzed the GnRH neuron system in *Prdm13–/–* mutants. Expression of *prdm13* has been reported in the olfactory placode that gives rise to GnRH neurons in zebrafish (41). We detected *Prdm13* expression by RT-PCR in both the mouse nasal compartment and the forebrain during developmental stages relevant to GnRH neuron migration (Figure 2A). Immunostaining revealed normal numbers of GnRH neurons in *Prdm13* mutants (GnRH neuron number mean ± SEM: *Prdm13+/+* 1341.33 ± 41.58 vs. *Prdm13–/–* 1453.00 ± 37.03; *P* = 0.1154, 2-tailed unpaired Student’s *t* test, *n* = 3 for each group). Similar numbers of GnRH-positive cells were observed in the nose, nasal-forebrain junction (nfj), and medial preoptic area (mpoa) of E14.5 mutants compared with WT mice (Figure 2, B–G; GnRH neuron number mean ± SEM: nose: *Prdm13+/+* 406.33 ± 32.10 vs. *Prdm13–/–* 431.33 ± 7.84, *P* = 0.4914; nfj: *Prdm13+/+* 378.67 ± 24.36 vs. *Prdm13–/–* 413.67 ± 27.63, *P* = 0.3958; mpoa: *Prdm13+/+* 556.33 ± 92.35 vs. *Prdm13–/–* 608.00 ± 10.02, *P* = 0.6077; 2-tailed unpaired Student’s *t* test, *n* = 3 for each group). GnRH neuron development is reflected postnatally in an innervation of the median eminence, the region where GnRH neurons release GnRH into the portal blood vessels of the pituitary. A comparison of GnRH neuron projections to the median eminence in P22 *Prdm13–/–* and *Prdm13+/+* littermates found no differences between the 2 genotypes (Figure 2, H and I). Together, these data suggest that *Prdm13*, although expressed during GnRH neuron development, does not play a prominent role in controlling the number or migration of these neurons.

### Prdm13 loss affects the development of Kiss1 neurons in the hypothalamus.

**Figure 3:**
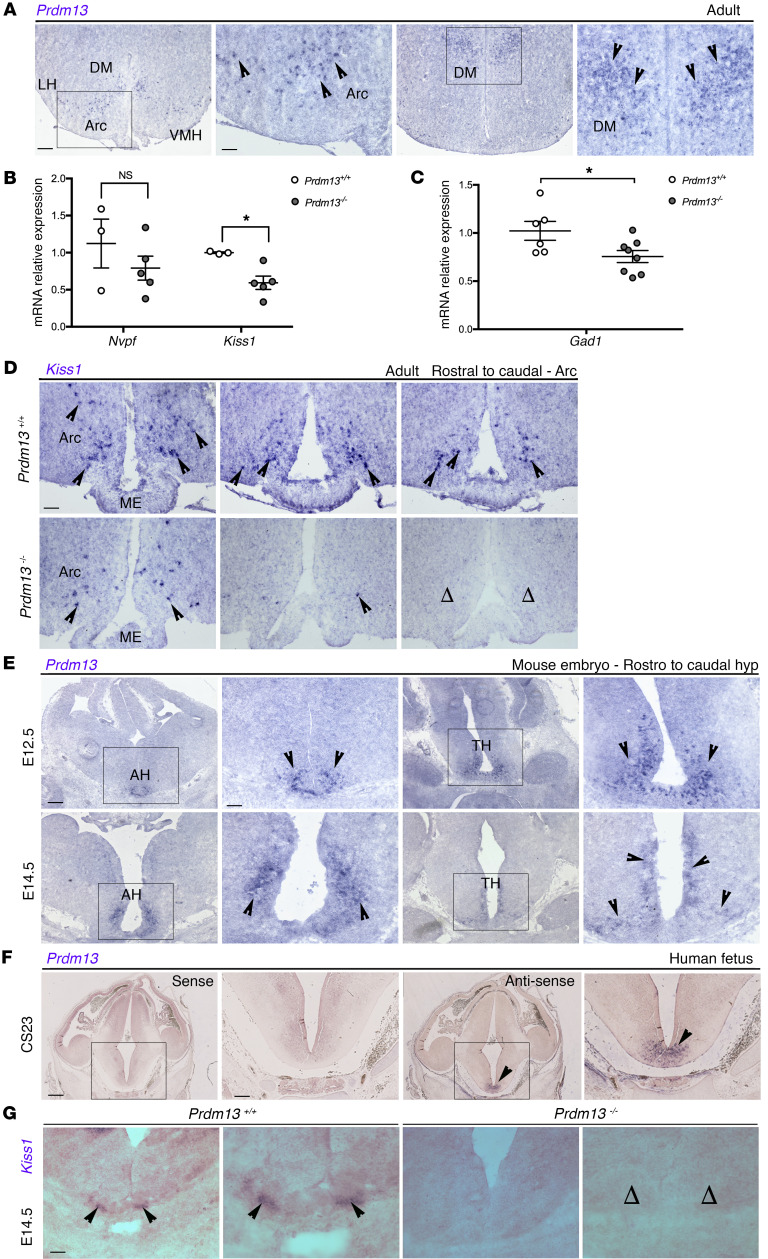
*Prdm13* loss affects *Kiss1* expression and *Kiss1* neuron development. (**A**) In situ hybridization on coronal adult WT male mice brain sections to detect *Prdm13* expression in the Arc and DM nuclei of the hypothalamus, indicated by black arrowheads. (**B**) qRT-PCR analysis for *Npvf* and *Kiss1* transcripts in the hypothalamus of *Prdm13+/+* and *Prdm13–/–* male mice. ΔΔCq was calculated relative to control samples using Cq threshold values normalized to the housekeeping gene *Gapdh*. Note the significant decrease of *Kiss1* levels in mutants. *P < 0.05, 2-tailed unpaired Student’s *t* test. (**C**) qRT-PCR analysis for *Gad1* transcripts in the hypothalamus of *Prdm13+/+* and *Prdm13–/–* from both sexes. ΔΔCq was calculated relative to control samples using Cq threshold values that were normalized to the housekeeping gene *Gapdh*. Note the significant decrease of *Gad1* levels in mutants. *P < 0.05, 2-tailed unpaired Student’s *t* test. (**D**) In situ hybridization on coronal sections from the Arc nucleus level from *Prdm13+/+* and *Prdm13–/–* male mice, detecting *Kiss1* transcripts. Note the reduction in *Kiss1* expression in the mutants compared with WT controls, where open arrowheads indicate complete absence of expression. (**E** and **F**) In situ hybridization on coronal sections detecting *Prdm13/PRDM13* expression in the developing mouse hypothalamus at E12.5 and E14.5 (**E**) and developing human hypothalamus at CS23 (**F**). mRNA transcripts are indicated by the arrowheads. The sense probe showed negative staining (first 2 images from the left). (**G**) In situ hybridization on coronal sections from E14.5 mouse embryo to detect *Kiss1* expression in *Prdm13+/+* and *Prdm13–/–*. Black arrowheads indicate examples of Kiss1-expressing cells; note the absence of Kiss1 neurons in mutants (open arrowheads). Areas within marked rectangles are shown in high magnification in adjacent images. Scale bars: 500 μm (**A**, **E**, and **F**, low magnification), 250 μm (**A**, **E**, and **F**, high magnification; **D** and **G**). LH, lateral hypothalamus; VMH, ventromedial hypothalamus; AH, anterior hypothalamus; TH, tuberal hypothalamus.

Microarray data have shown that *Prdm13* expression is enriched in the adult dorso-medial (DM) and, to a lesser extent, in the arcuate (Arc) nuclei (42) of the hypothalamus, where GnIH and Kiss1 neurons are found, respectively. Thus, we asked whether GnRH deficiency might be indirectly caused by the lack or malfunctioning of hypothalamic neurons that regulate GnRH secretion. First, we visualized *Prdm13* expression in the adult brain by in situ hybridization, which confirmed *Prdm13* expression in the Arc and DM nuclei of the adult hypothalamus (Figure 3A).

We then assessed whether *Prdm13* could regulate *Npvf* (which encodes GnIH) and *Kiss1* expression in the adult male hypothalamus. qRT-PCR analyses showed a marked reduction of *Kiss1* mRNA levels in *Prdm13–/–* mice compared with *Prdm13+/+* mice. *Npvf* expression appeared slightly reduced, but this difference was not significant (Figure 3B). Because PRDM13 is known to control GABAergic neuronal cell fate in several brain areas (33–35) and GABAergic neurotransmission plays important roles in sustaining fertility (43), we also analyzed *Gad1* expression and found a significant decrease of *Gad1* levels in *Prdm13–/–* mice compared with *Prdm13+/+* mice (Figure 3C). However, the expression of typical GABAergic hypothalamic neuronal markers, such as *Pomc* and *Npy/Agrp* (44), was similar in *Prdm13–/–* and *Prdm13+/+* mice (Supplemental Figure 2A), suggesting that only a subset of hypothalamic neurons requires PRDM13.

Next, we visualized *Kiss1*-expressing neurons in adult male mouse brains by in situ hybridization. This analysis, although qualitative, clearly revealed fewer *Kiss1*-expressing cells in the Arc nucleus of *Prdm13–/–* mice compared with *Prdm13+/+* controls (Figure 3D). Kiss1 neurons can be found in the anteroventral periventricular (AVPV) nucleus of the hypothalamus as well (45), where they contribute to GnRH secretion, especially in females, by increasing their activity during the GnRH/LH surge (46). *Prdm13* transcripts could not be detected in the AVPV nucleus of P15 and adult brains (Supplemental Figure 2B). Accordingly, normal numbers and distribution of *Kiss1*^+^ neurons were observed in the AVPV nucleus of *Prdm13–/–* mutants (Supplemental Figure 2, C and D; *Kiss1*^+^ neuron number mean ± SEM: *Prdm13+/+* 238.3 ± 38.62 vs. *Prdm13–/–* 218.3 ± 8.67; *P* = 0.6399, 2-tailed unpaired Student’s *t* test, *n* = 3 for each group). Consistent with this finding, we could not detect significantly reduced *Kiss1* expression in whole female *Prdm13–/–* hypothalami, presumably due to the high numbers of *Kiss1+* AVPV neurons in females (ref. 46 and Supplemental Figure 2E).

To determine whether PRDM13 had a role in the development of Kiss1 neurons, we further examined the expression of *Prdm13/PRDM13* in the developing mouse and human hypothalamus. *Prdm13* is expressed in the preoptic and tuberalis nuclei of the developing mouse hypothalamus (Figure 3E). We also detected prominent *PRDM13* expression in the developing hypothalamus of a human fetus at Carnegie stage (CS) 23 (Figure 3F). The first *Kiss1-*expressing cells can be detected as early as E13.5 near the third ventricle of the embryonic mouse brain (47), with numbers increasing as development proceeds. Thus, we visualized Kiss1 neurons in the brains of E14.5 mice by in situ hybridization and found that the number of these cells in the presumptive Arc nucleus of *Prdm13–/–* embryos was significantly reduced compared with that in WT littermates (Figure 3G; *Kiss1*^+^ neuron number mean ± SEM: *Prdm13+/+* 79.33 ± 8.37 vs. *Prdm13–/–* 0.00; *P* = 0.0007, 2-tailed unpaired Student’s *t* test, *n* = 3 for each group). To obtain insights into the possible mechanisms through which PRDM13 affects the number of Kiss1 neurons, we compared cell proliferation and apoptosis in the developing hypothalamus between E14.5 *Prdm13+/+* and *Prdm13–/–* embryos via immunodetection of phosphohistone 3B–positive (PH3B-positive) and cleaved caspase-3–positive (CC3-positive) cells. No significant differences in the numbers of PH3B^+^ and CC3^+^ cells were observed between the genotypes (Supplemental Figure 3), suggesting a differentiation defect rather than reduced precursor proliferation or increased cell death.

Together, these findings demonstrate that PRDM13 is required for the development of *Kiss1*-expressing neurons in the Arc nucleus of the hypothalamus. As Kiss1 promotes GnRH secretion, a deficiency in Kiss1 neurons in patients with a homozygous *PRDM13* mutation may underlie the human CHH phenotype.

### Prdm13-null mice display normal gonadal structure, but delayed vaginal opening.

**Figure 4:**
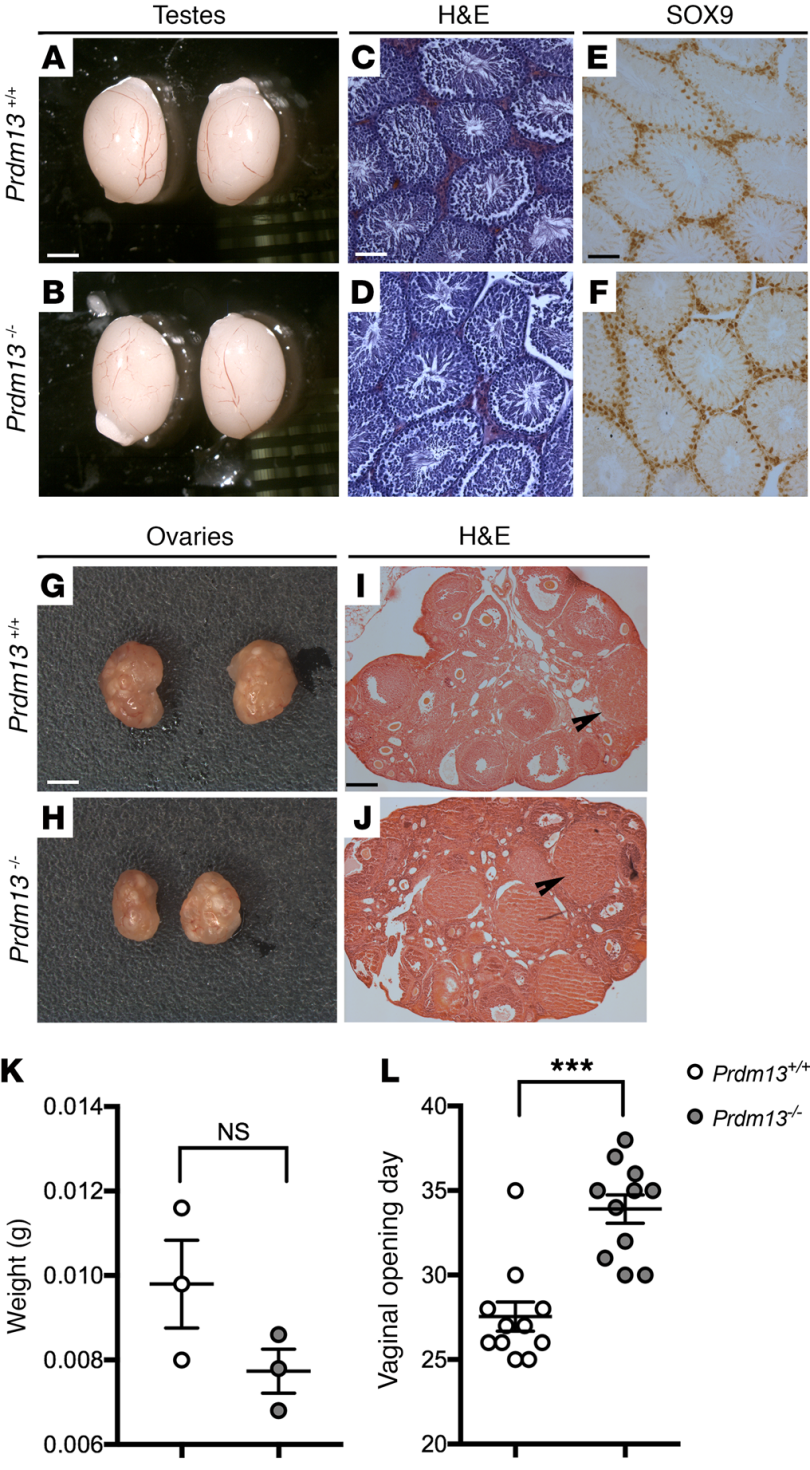
*Prdm13*-deficient mice display normal gonadal structure, but delayed vaginal opening. (**A** and **B**) Microphotograph of testis pairs of the indicated genotypes; no differences were observed in their size. (**C**–**F**) H&E-stained (**C** and **D**) and SOX9-immunostained (**E** and **F**, Sertoli cells marker) testis representative sections from adult male mice of indicated genotypes. No differences and normal spermatogenesis were observed in WT and mutant mice. (**G** and **H**) Microphotograph of ovary pairs of the indicated genotypes. (**I** and **J**) H&E-stained ovary representative sections from mice of indicated genotypes. No differences in corpora lutea number were observed between WT and mutant mice. (**K**) Weight of ovaries from adult female mice. No differences were observed between WT and mutant mice (2-tailed unpaired Student’s *t* test). (**L**) Age at the time of the vaginal opening of the indicated genotypes. Note the significant delay in vaginal opening of *Prdm13–/–* female mice. ***P < 0.001, 2-tailed unpaired Student’s *t* test. Scale bars: 1.5 mm (**A**, **B**, **G**, and **H**); 250 μm (**C**–**F**); 500 μm (**I** and **J**).

The loss of kisspeptin can result in abnormal sexual maturation, with significant phenotypic variability (48). An examination of testis size and morphology of adult postpubertal *Prdm13–/–* mice did not detect significant differences in gonadal size between P60 *Prdm13–/–* mice and WT littermates (Figure 4, A and B). Furthermore, *Prdm13–/–* mice displayed normal spermatogenesis, as assessed by H&E staining and immunodetection of the Sertoli marker SOX9, which, together with SOX8, is essential for testis formation and spermatogenesis (ref. 49 and Figure 4, C–F). We also analyzed the gonads of adult *Prdm13–/–* females. The ovaries appeared histologically normal (Figure 4, G–J) and did not display a significant reduction in weight (Figure 4K; weight mean ± SEM = *Prdm13+/+* 0.0098 ± 0.001 g vs. *Prdm13–/–* 0.0077 ± 0.00005 g; *P* = 0.15, 2-tailed unpaired Student’s *t* test, *n* = 3 for each group). Yet a significant delay in the timing of vaginal opening was observed in *Prdm13–/–* females compared with *Prdm13+/+* controls (Figure 4L; vaginal opening day mean ± SEM = *Prdm13+/+* 27.55 ± 0.87 vs. *Prdm13–/–* 33.91 ± 0.84; *P* < 0.0001, 2-tailed unpaired Student’s *t* test, *n* = 11 for each group). These observations suggest that the reduction in *Kiss1* neurons in *Prdm13–/–* mice was not sufficient to cause a gross gonadal phenotype, but still resulted in delayed pubertal onset.

### Prdm13 expression in the human and mouse cerebellar VZ.

**Figure 5:**
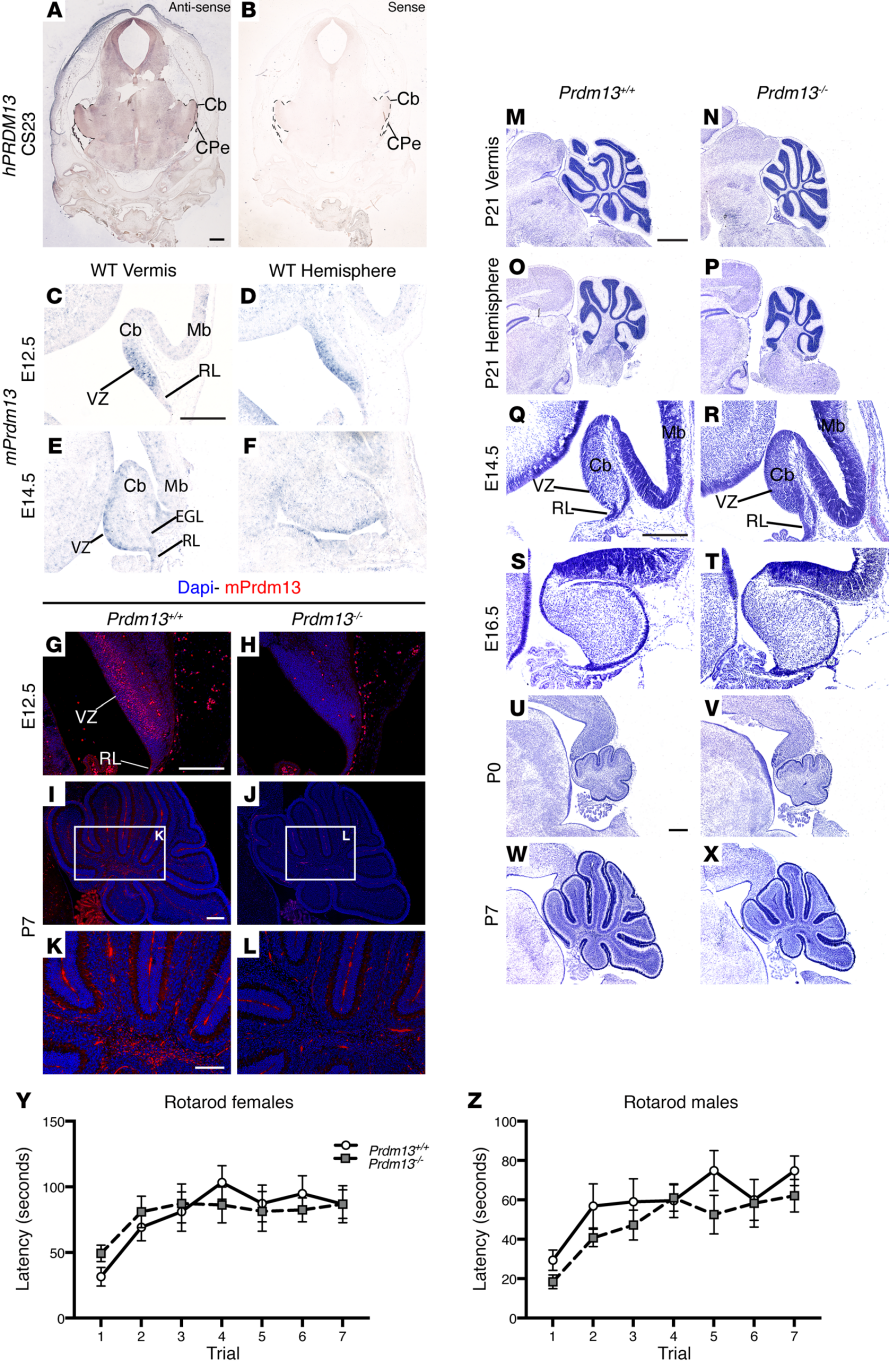
*PRDM13* has a conserved role in regulating cerebellar growth during development. (**A**) In situ hybridization for human *PRDM13* (*hPRDM13*) transcripts (brown) in a coronal section through the cerebellum at CS23. (**B**) The *hPRDM13* sense control showed no *PRDM13* transcript staining throughout the developing cerebellum. (**C**–**F**) In situ hybridization for *Prdm13* exon 4 transcripts (blue) in sagittal sections through the vermis and hemisphere of *Prdm13+/+* cerebella at stages indicated. (**G**–**J**) Immunohistochemistry of sagittal sections of *Prdm13+/+* and *Prdm13–/–* cerebella using PRDM13 antiserum at indicated stages. High-power images are shown at P7 (**K** and **L**). Note that *Prdm13* transcripts (**C**–**F**) and protein (**G**) are predominantly restricted to the VZ at midembryonic stages and to the cerebellar white matter postnatally (**I**, **K**). (**M**–**P**) Cresyl violet–stained sagittal sections through the cerebellum of P21 *Prdm13+/+* and *Prdm13–/–* mice, anterior to the left. Note hypoplasia of the cerebellar vermis and hemispheres in *Prdm13–/–* mice. (**Q**–**X**) Time course through cerebellar development using Cresyl violet–stained sagittal sections of the cerebellar vermis of *Prdm13+/+* and *Prdm13–/–* mice. Note that cerebellar hypoplasia is clearly evident in postnatal stages in *Prdm13–/–* mice. (**Y** and **Z**) Mean latency of female (**Y**) and male (**Z**) mice to remain on the rotarod over the course of 7 trials (*n* = 10 of each sex and genotype). Note that there is no difference between genotypes in female or male mice (2-way repeated measures ANOVA for genotype and trial). RL, rhombic lip; Mb, midbrain; Cb, cerebellum; CPe, choroid plexus. Scale bars: 600 μm (**A**); 300 μm (**C**, **G**, **I**, **K**, **Q**, and **U**); 1 mm (**M**). Disclosure: The images in Figure 5, O, P, W, and X are presented again in Supplemental Figure 4, W, X, Q, and R, respectively.

To understand how *PRDM13* deficiency can lead to cerebellar hypoplasia, we first examined *PRDM13* expression during development. *PRDM13* was expressed in the CS23 human cerebellar primordium (Figure 5, A and B). Similarly, *Prdm13* expression was observed in the E12.5–E14.5 mouse cerebellum, with *Prdm13* transcripts detected prominently in the VZ of both the cerebellar vermis and hemispheres (Figure 5, C–F). Immunostaining with antiserum raised against a C-terminal fragment of PRDM13 (33) confirmed the presence of PRDM13 in the E12.5 cerebellar anlage (Figure 5G). At postnatal stages, PRDM13 localized to cells in the prospective white matter (Figure 5, I and K). The specificity of immunostaining was confirmed by the absence of staining in the cerebellum of *Prdm13–/–* mice (Figure 5, H, J, and L).

### Prdm13 is required for normal postnatal cerebellar growth.

An examination of cerebellar structure and development revealed hypoplasia of the cerebellar vermis and hemispheres of *Prdm13–/–* mice at P21, when cerebellar development is largely complete (Figure 5, M–P). To identify the onset of reduced cerebellar growth in *Prdm13* mutants, cerebellar development was analyzed from E14.5 to P7 (Figure 5, Q–X, and Supplemental Figure 4). Cerebellar surface area was not significantly altered at birth in the cerebellar vermis (mean ± SEM = 0.51 ± 0.02 mm^2^ in *Prdm13+/+*, 0.51 ± 0.03 mm^2^ in *Prdm13–/–*; *P* = 0.9259, 2-tailed unpaired Student’s *t* test, *n* = 4 for each group) or cerebellar hemispheres (mean ± SEM = 0.69 ± 0.02 mm^2^ in *Prdm13+/+*, 0.54 ± 0.09 mm^2^ in *Prdm13–/–*; *P* = 0.1181, 2-tailed unpaired Student’s *t* test, *n* = 4 for *Prdm13+/+* and *n* = 3 for *Prdm13–/–* mice). Analysis at later stages revealed that the onset of the phenotype differed between cerebellar regions. By P5, hypoplasia was evident in the vermis (mean ± SEM = 1.71 ± 0.02 mm^2^ in *Prdm13+/+*, 1.27 ± 0.04 mm^2^ in *Prdm13–/–*; *P* = 0.0007, 2-tailed unpaired Student’s *t* test, *n* = 3 for each group), while hemisphere size did not significantly differ between genotypes at P5 (mean ± SEM = 1.32 ± 0.17 mm^2^ in *Prdm13+/+*, 1.17 ± 0.06 mm^2^ in *Prdm13–/–*; *P* = 0.4131, 2-tailed unpaired Student’s *t* test, *n* = 3 for each group) (Supplemental Figure 4, M–P). By P7, cerebellar hypoplasia was, however, clearly evident in both the cerebellar vermis (mean ± SEM = 2.63 ± 0.09 mm^2^ in *Prdm13+/+*, 1.95 ± 0.07 mm^2^ in *Prdm13–/–*; *P* = 0.0049, 2-tailed unpaired Student’s *t* test, *n* = 3 for each group) and hemispheres (mean ± SEM = 2.40 ± 0.18 mm^2^ in *Prdm13+/+*, 1.80 ± 0.09 mm^2^ in *Prdm13–/–*; *P* = 0.0377, 2-tailed unpaired Student’s *t* test, *n* = 3 for each group) (Figure 5, W and X, and Supplemental Figure 4, Q–T). To investigate the mechanism through which *Prdm13* deficiency leads to cerebellar hypoplasia, proliferation and cell death were assessed at E16.5, P0, and P5 prior to and at the onset of the phenotype. Proliferating PH3B^+^ and apoptotic CC3^+^ cells in the external germinal layer (EGL), where glutamatergic granule neuron progenitors expand, and the rest of the cerebellum were counted separately. Proliferation was not significantly altered in either region of *Prdm13* mutants (Supplemental Figure 5, A–D). In contrast, the number of apoptotic cells was significantly increased in non-EGL cells in both the vermis and hemispheres of *Prdm13*-deficient cerebella at P0 (Supplemental Figure 5, F, H, J, L, N, and P). Together, these data indicate that *Prdm13* is required for normal postnatal cerebellar development and that reduced growth in *Prdm13–/–* mutants occurs, at least in part, due to increased apoptosis of cells outside the EGL.

To determine whether the cerebellar hypoplasia in these mutants is associated with motor coordination and learning deficits, we tested mice on an accelerating rotarod. Adult *Prdm13–/–* mice of both sexes exhibited normal motor coordination and motor learning in this test (Figure 5, Y and Z), suggesting that the cerebellar changes in *Prdm13–/–* mutants were not sufficient to cause overt motor deficits.

### Prdm13 regulates GABAergic cell-fate determination in the developing cerebellum.

As *Prdm13* expression was largely confined to cerebellar GABAergic neuron progenitor zones in the embryonic cerebellum (Figure 5, A–F), the number and distribution of the 2 major GABAergic lineage (PAX2^+^ interneurons and LHX1/5^+^ PC) progenitors were assessed. PC progenitors were visualized at 3 key stages of development: E14.5, when cerebellar corticogenesis is initiated by formation of the Purkinje plate (PP), a transient structure comprising several layers of PCs; P0, just prior to PC monolayer formation; and P7, when the PC monolayer has formed. Analysis revealed normal PP formation in *Prdm13–/–* mice (Supplemental Figure 6, A–H) with no apparent alteration in PC distribution and organization at birth (Supplemental Figure 6, I–P) or alteration in the PC monolayer postnatally (Supplemental Figure 6, Q–T). These findings suggest that *Prdm13* is not required for PC formation, migration, and organization. To investigate whether *Prdm13* influences PC differentiation, dendritogenesis was analyzed using molecular layer thickness as a measure of dendritic span (50). No significant difference was detected between genotypes. These findings suggested that PC differentiation was unaffected by *Prdm13* deficiency, but we cannot completely exclude subtle abnormalities at this stage (Supplemental Figure 6, U–Y).

**Figure 6:**
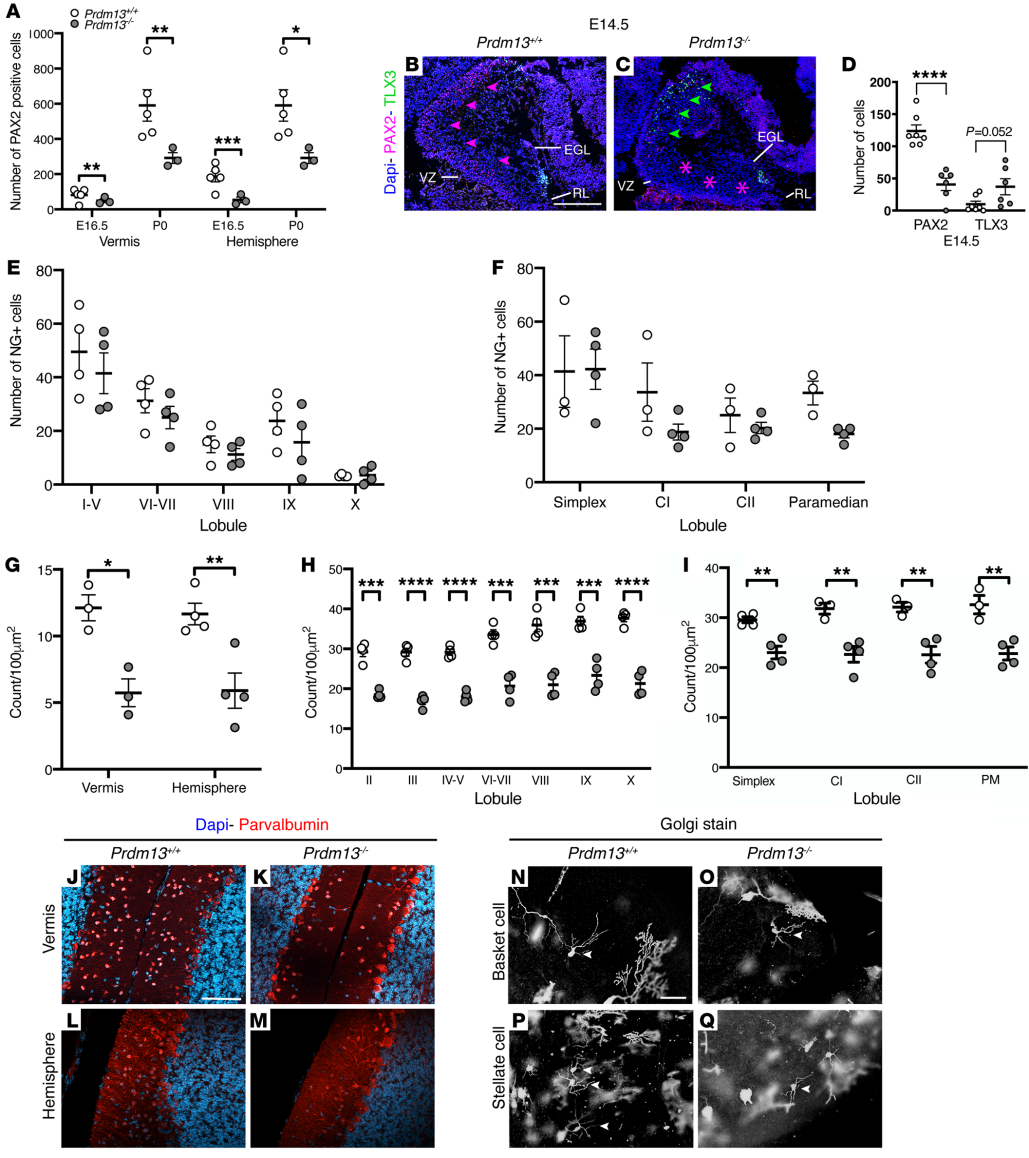
PRDM13 is a critical regulator of GABAergic cell fate in the cerebellum. (**A**) PAX2 cell counts in *Prdm13+/+* and *Prdm13–/–* cerebella. Note the reduction in PAX2^+^ cells in the vermis and hemisphere of *Prdm13–/–* mice at E16.5 and P0 (*n* = 4 per genotype). (**B** and **C**) Immunohistochemistry of sagittal cerebella sections at E14.5 of *Prdm13+/+* and *Prdm13–/–* mice using antibodies to PAX2 and TLX3 to label GABAergic interneurons and glutamatergic progenitors, respectively. Note the marked reduction in PAX2^+^ cells in *Prdm13–/–* mice (**C**) (pink asterisks) and the concomitant increase in TLX3^+^ cells (**C**) (green arrowheads). (**D**) Quantification of PAX2^+^ and TLX3^+^ cells is shown at E14.5. Note the decrease in PAX2^+^ cells, accompanied by an increase in TLX3^+^ neurons. (**E** and **F**) Neurogranin^+^ Golgi cell counts in *Prdm13+/+* and *Prdm13–/–* vermis (**E**) and hemispheres (**F**) at P21. Note the similar number of Golgi cells between genotypes. (**G**) Quantification of parvalbumin^+^ MLIs in the cerebellum at P21. Note the reduction in MLIs in the vermis and hemispheres of *Prdm13–/–* mice. (**H** and **I**) Lobule-specific MLI cell counts in the vermis and hemispheres at P21. Note the significant reduction in MLIs across all vermis and hemisphere lobules of *Prdm13–/–* cerebella. (**J**–**M**) Immunohistochemistry of sagittal cerebella sections at P21 of *Prdm13+/+* and *Prdm13–/–* mice using antibodies to parvalbumin to label MLIs. Note the reduction in parvalbumin^+^ cells in the vermis (**K**) and hemispheres (**M**) of *Prdm13–/–* mice. (**N**–**Q**) Golgi-Cox–stained sagittal sections of adult *Prdm13+/+* and *Prdm13–/–* cerebella. Note that the morphology of the basket (**N** and **O**) and stellate cells (**P** and **Q**) is consistent between *Prdm13+/+* and *Prdm13–/–* mice. *P < 0.05; **P < 0.01; ***P < 0.001, 2-tailed unpaired Student’s *t* test. NG, neurogranin; CI, Crus I; CII, Crus II. Scale bars: 300 μm (**B**); 100 μm (**J** and **N**).

Next, the PAX2^+^ population was visualized from the onset of their specification (51, 52). At E12.5, PAX2^+^ cells were present in a small cluster in the lateral cerebellum in WT mice (Supplemental Figure 7, A–D), but absent from *Prdm13–/–* cerebella (Supplemental Figure 7, E–H). At E14.5, PAX2^+^ cells were present as a thin layer throughout the dorsal-ventral aspect of the VZ along the entire mediolateral extent of the cerebellum in *Prdm13+/+* mice (Supplemental Figure 7, Q–T). In contrast, no PAX2^+^ cells were present in the dorsal region of the VZ in *Prdm13–/–* mice, with only a few cells present in the most ventral aspect of the cerebellum (Supplemental Figure 7, U–X). Quantification of PAX2^+^ cells at subsequent stages of development indicated that the number of PAX2^+^ progenitors was significantly reduced in *Prdm13–/–* cerebella (Figure 6A). A previous study showed that *Prdm13* deficiency in the spinal cord is associated with a reduction in GABAergic PAX2^+^ progenitors and an accompanying increase in glutamatergic progenitors (35). To determine whether PRDM13 has a similar role in suppressing glutamatergic TLX3^+^ cell fate in the developing cerebellum, TLX3^+^ progenitors were visualized by immunostaining. At E12.5, TLX3^+^ cells were restricted to the most ventral aspect of the cerebellum in all regions apart from the most lateral cerebellum, where they were completely absent (Supplemental Figure 7, I–L). TLX3^+^ cells expanded dorsally in *Prdm13–/–* mutants to occupy the majority of the dorsal-ventral extent of the cerebellum, almost extending to the level of the rhombic lip. These findings are identified across the entire mediolateral cerebellum in *Prdm13–/–* mutants (arrowheads, Supplemental Figure 7, M–P). At E14.5, few TLX3^+^ neurons are normally present outside of the EGL in WT mice (Figure 6B). TLX3^+^ cells were clearly present in the *Prdm13*-deficient cerebellum just outside the VZ where PAX2^+^ cells are normally found (Figure 6C). Quantification confirmed the reduction in PAX2^+^ progenitors and a concomitant increase in TLX3^+^ cells (Figure 6D). We subsequently asked whether the misspecified TLX3^+^ or PAX2^+^ cells accounted for the increase in cell death in the non-EGL mutant cerebella at birth. No apoptotic PAX2^+^ or TLX3^+^ cells were identified, suggesting that *Prdm13* is not required for the survival of these cell types in the newborn cerebellum. Together, these data identified a role for PRDM13 in the specification of GABAergic PAX2^+^ cells by suppressing glutamatergic cell fate.

The cerebellar cortex comprises 3 distinct layers where highly stereotyped connections between neurons form a repeating and relatively simple cerebellar circuit. PAX2^+^ precursors form a diverse population of GABAergic interneurons, which integrate into and regulate the output of all levels of the cerebellar circuit through local inhibition. To determine the impact of PAX2^+^ misspecification in *Prdm13–/–* mutants, we analyzed the 2 major GABAergic interneuron populations of the cerebellar cortex, Neurogranin^+^ Golgi cells and Parvalbumin^+^ molecular layer interneurons (MLIs). Golgi cells were distributed appropriately throughout the granular cell layer with normal cell numbers in all lobules of the cerebellar vermis and hemispheres of *Prdm13*-deficient cerebella (Figure 6, E and F). In contrast, MLI cell number, confined to the molecular layer of the cerebellar cortex, was significantly reduced in *Prdm13–/–* mice (Figure 6, G and J–M). A finer analysis revealed that this reduction was uniform across all cerebellar lobules of the vermis and hemispheres (Figure 6, H and I). Golgi-Cox staining was used to visualize 2 recognized subtypes of MLIs, basket and stellate cells. Small somata of basket and stellate interneurons were respectively identified in the inner and outer portions of the molecular layer (Figure 6, N–Q). No morphological change in these neurons was observed in *Prdm13–/–* mutants. Specifically, basket cells had long, straight dendrites that extended toward the pial surface with a single, main axon that ran parallel to the PC layer (Figure 6O). Stellate cells were identifiable by their characteristic star-like appearance, created by radiation of dendrites from the cell body (Figure 6Q).

Together, these findings suggest that misspecification of PAX2^+^ cells results in fewer MLIs in the adult cerebellum. As MLIs are postulated to influence cerebellar-regulated behaviors by regulating PC activity (50, 53–55), deficits in MLIs may underlie some of the neurological symptoms observed in individuals with homozygous *PRDM13* mutations.

## Discussion

Here we describe a recessive syndrome most likely caused by a hypomorphic mutation of *PRDM13* that is present at low levels in the Maltese population and inherited from unaffected, heterozygous parents. Patients homozygous for this mutation exhibited a constellation of phenotypes that included developmental delay, CHH, cerebellar hypoplasia, scoliosis, and intellectual disability. To understand how *PRDM13* deficiency might lead to CHH and cerebellar hypoplasia, we studied hypothalamic and cerebellar development in a homozygous *Prdm13*-deficient mouse model. This analysis identified key roles for PRDM13 in the development of Arc Kiss1 neurons in the hypothalamus and MLIs in the cerebellum. Intriguingly, both these neuronal populations also require the transcription factor PTF1A for normal development, and since PTF1A has been shown to control *Prdm13* expression in the developing cerebellum and spinal cord, our findings suggest that the CHH and cerebellar phenotypes are functionally linked by central roles for PTF1A and PRDM13 in neuronal cell-fate specification in both tissues.

### Recessive PRDM13 mutation is associated with a unique combination of phenotypes.

The combination of developmental delay, CHH, cerebellar hypoplasia, scoliosis, and intellectual disability associated with *PRDM13* mutation differs significantly from what is seen in other conditions with CHH and cerebellar atrophy, such as Gordon Holmes syndrome (GHS). The latter is an autosomal recessive adult-onset neurodegenerative disorder characterized by progressive cognitive decline, dementia, and variable movement disorders, such as ataxia and chorea. CHH and progressive cerebellar ataxia may also be prominent features in Boucher-Neuhäuser, Oliver-McFarlane, and Lawrence-Moon syndromes (56–58). In contrast with the late-onset neurodegenerative etiology of cerebellar atrophy in these conditions, we show here that *PRDM13* mutations disrupt cerebellar development.

### The PTF1A-PRDM13 axis in Kiss1 neuron development.

Previous studies showed that PRDM13 functions downstream of PTF1A, which plays distinct roles in the regulation of both the cerebellum and the hypothalamus (18, 19, 38). Our observations support the existence of overlapping phenotypes between *Prdm13*- and *Ptf1a*-deficient mice in the hypothalamus and cerebellum, implying that transcriptional dysregulation in specific neuronal progenitors is the most likely pathogenic mechanism underlying the phenotypes associated with *PRDM13* mutation. Although *PTF1A* mutations have been linked to cerebellar aplasia and hypoplasia, no link to CHH in humans has, to our knowledge, been reported to date. Defective Kiss1 neuron development has been described in mice with conditional forebrain-specific *Ptf1a* deletion using the Nkx2.1-Cre driver (38). Interestingly, in this study, Kiss1 neurons were not found to be in the *Ptf1a* lineage. Thus, PTF1A induction of *Prdm13* expression in the same lineage cells could instruct neighboring cells to differentiate into Kiss1 neurons via transmembrane and/or secreted proteins (38, 59). Members of the semaphorin family were among the genes affected by PTF1A loss in the forebrain (38). Given their key roles in the control of the reproductive axis (60), it will be interesting to explore their possible involvement in the differentiation program of Kiss1 neurons, thus shedding light onto the cellular mechanisms through which the PTF1A/PRDM13 axis regulates reproduction.

Our data highlight that PRDM13 selectively controls a subset of hypothalamic neurons, the Arc Kiss1 neurons, underlining a cell-specific role of PRDM13 in the differentiation program of the hypothalamus. Notably, these neurons are mainly glutamatergic, thus revealing a role for PRDM13 in the hypothalamus different from that in the retina and spinal cord (33–35), where it controls GABAergic cell-fate decision. This is in agreement with previous findings showing that the upstream PTF1A transcription factor in the hypothalamus does not control the GABAergic:glutamatergic balance, but rather the cell fate of specific neuronal populations (38). However, despite an overall downregulation of *Gad1* in the hypothalamus of *Prdm13*-deficient mice, the expression of typical hypothalamic GABAergic neuronal markers was unaltered. Yet given that GABAergic neurotransmission at the hypothalamic level is on its own fundamental in sustaining fertility and pubertal onset (43), reduced levels of GABA might also contribute to CHH observed in the patients.

Interestingly and in contrast with what is shown in *Ptf1a*-deficient mice, our data also indicate that *Prdm13* deficiency specifically affects Kiss1 neurons in the Arc nucleus, but not in the AVPV, suggesting a highly restrictive role for PRDM13 in the specification of Arc Kiss1 neurons and the possible involvement of additional transcription factors in the specification of AVPV Kiss1 neurons. These observations may also explain the inability to detect a significant reduction in *Kiss1* expression in the whole hypothalamic region of female *Prdm13*-deficient mice (Supplemental Figure 2E), as unaffected AVPV Kiss1 neurons are more dense in females than in males (46).

While patients carrying *PRDM13* mutations present with CHH, we did not find typical phenotypes of hypogonadism in *Prdm13*-deficient mice, except for a delayed pubertal onset in female mice. Importantly, although we were not able to detect Kiss1 neurons in E14.5 *Prdm13*-deficient mice, some Kiss1 neurons were present in the Arc nucleus of adult *Prdm13*-deficient mice. Previous studies have shown that a residual 5% function of Kiss1 neurons is sufficient to guarantee male mouse fertility (61) and that mice lacking *Kiss1* display a variable reproductive phenotype (48). Thus, our data suggest that sufficient numbers of Kiss1-expressing cells differentiate in *Prdm13–/–* mutants to support normal sexual development, whereas the reduced number may have an impact on pubertal onset, causing a delay.

To our knowledge, this is the first evidence linking disrupted PRDM13-mediated regulation of Kiss1 neurons to CHH in humans. It would be interesting to assess the effects of Kisspeptin administration on gonadotropin secretion in our mouse model, leading to a potential treatment for patients that carry a *PRDM13* mutation.

### PRDM13 as a key GABAergic fate regulator in the cerebellum.

The molecular mechanisms that control the specification, maintenance, and differentiation of GABAergic progenitors from a common PTF1A^+^ progenitor pool in the cerebellar VZ remain ill defined. We have identified a role for PRDM13 in controlling GABAergic fate in the developing cerebellum. As *Prdm13* expression requires PTF1A in the cerebellum (35), we conclude that PRDM13 functions as a critical effector protein downstream of PTF1A in GABAergic cell-fate regulation. Specifically, we demonstrate a differential requirement for PRDM13 in GABAergic neuronal development, where PRDM13 is necessary to generate a subset of PAX2^+^ GABAergic interneurons, but appears largely dispensable for PC development. The relatively mild phenotype in *Prmd13* mutants is in contrast with the cerebellar agenesis associated with *Ptf1a* deficiency (18). It will be of interest to assess cerebellar and hypothalamic phenotypes in *Prdm13* conditional mutants to determine whether the milder phenotypes in our *Prdm13* mutants occur because PTF1A regulates other genetic pathways in addition to *Prdm13* or whether the milder phenotype is merely a consequence of residual PRDM13 function in our *Prdm13* mutants.

More broadly, these data implicate PRDM13 in the regulation of excitatory/inhibitory balance in the cerebellum. GABAergic PAX2^+^ interneuron progenitors are lost in *Prdm13*-deficient mice, and progenitors expressing the glutamatergic marker TLX3 appear to take their place, which is similar to findings in *Ptf1a* mutants (23). Cerebellar dysfunction and altered cerebello-cortical circuitry is thought to contribute substantially to complex neuropsychiatric diseases and has been linked to autism (62), intellectual disability, and schizophrenia (63, 64). One pathophysiological theory linking these genetically heterogenous and diverse neuropsychiatric disorders is of altered excitatory/inhibitory balance (65), a theory that has been gaining significant traction. The observation that patient 3 also developed epileptic seizures adds further weight to the possibility of altered excitatory/inhibitory balance in *PRDM13* syndrome.

To conclude, we have identified what we believe to be a novel, recessive syndrome associated with a mutation in the *PRDM13* gene. Our analysis of mice homozygous for a hypomorphic *Prdm13* allele suggests that the phenotypic association of CHH with cerebellar hypoplasia occurs as a result of the central function of PRDM13 in controlling GABAergic and Kiss1 neuronal specification and differentiation in the cerebellum and hypothalamus, respectively. Patients with recessive, hypomorphic *PRDM13* and/or *PTF1A* mutations are likely to be extremely rare. Our data showing that the 3 patients and the unrelated heterozygous carrier shared an identical 0.2 Mb region of homozygosity that encompassed the *PRDM13* mutation, which extended for 1.6 Mb in total in the 3 patients (Supplemental Table 3), suggest that the *PRDM13* mutation is present on a common haplotype within the Maltese population and may in turn lead to identification of further patients with this phenotype. The existence of patients with *PRDM13* mutations and the associated phenotypes reported here in *Prdm13*-deficient mice represent an important step toward dissecting the molecular mechanisms controlling cell-fate determination in the developing nervous system. These mechanisms are likely to contribute to excitatory/inhibitory imbalance in the brain, which may underlie a range of different neurodevelopmental phenotypes.

## Methods

### Whole exome and Sanger sequencing.

Whole exome capture was performed on all family members using Agilent SureSelect, version 4, according to the manufacturer’s protocol. Enriched libraries were sequenced on the Illumina HiSeq 2500. Sequencing reads passing quality filters were aligned to the reference genome build GRCh37/hg19 using the Burrows-Wheeler Aligner (BWA) algorithm, and for variant calling, we applied GATK (66) base quality score recalibration, indel realignment, and duplicate removal and performed SNP and INDEL discovery and genotyping using standard hard-filtering parameters or variant quality score (67). The variant annotation and interpretation analyses were generated using Ingenuity Variant Analysis (version 4.0.20151113) software from QIAGEN. Disease-causing variants were validated by Sanger sequencing.

### Exome sequencing filtering strategy.

Exonic and cryptic splice site variants (±5), which were homozygous in the affected siblings (patients 1 and 2) and heterozygous in the parents, had a call quality of 20 or more, read depth of 10 or more, and a frequency of less than 0.1% in public exome databases (gnomAD browser, 1000 genomes, NHLBI ESP exomes, ExAC) or were known pathogenic variants listed in the Human Genome Mutation Database (HGMD). Initially, Kallmann syndrome, hypogonadotropic hypogonadism (HH), cerebellar hypoplasia, developmental delay, or diseases consistent with these phenotypes were set as the biological terms in our filtering criteria.

### Microarray.

Four samples from the 3 affected patients and an unrelated heterozygous Maltese carrier of the 13 bp deletion in PRDM13 were genotyped using an Illumina Infinium OmniExpress-48 microarray according to the manufacturer’s instructions and processed using Illumina software.

### Mice.

*Prdm13* mutant mice lacking exons 2 and 3, which encode much of the PR domain, have been described and maintained on a C57BL/6 background (33). Mice were genotyped by PCR using DNA obtained from the ear as described in that original publication (see Supplemental Methods for primer sequences). All mice were maintained and bred in the Biological Services Unit, King’s College London, Guy’s Campus.

### Histology.

Samples were dissected in PBS and fixed in 4% paraformaldehyde overnight at 4°C and either cryopreserved in 30% sucrose for OCT embedding or, after dehydration, infiltrated and embedded in paraffin wax. For samples immunostained for TLX3, dissected samples were cryoprotected in 7.5% gelatin and 15% sucrose. Serial sagittal sections were cut at 10 μm before drying at 42°C overnight. Sagittal or coronal cryosections were cut at 10–20 μm using a cryostat.

### 0.1% Cresyl violet acetate staining.

Sections were deparaffinized in xylene, rehydrated through graded ethanol solutions, and stained with 0.1% Cresyl violet acetate (*Nissl*) for 10 minutes. Differentiation of the stain was achieved using glacial acetic acid. Stained sections were dehydrated, placed in xylene, and mounted.

### H&E staining.

Sections were deparaffinized in xylene and rehydrated in graded ethanols. Sections were stained with Erhlich’s hematoxylin for 10 minutes, washed to remove excess staining, and immersed in acid alcohol (0.5% HCl, 70% ethanol) for 15 seconds. Sections were then stained with 0.5% aqueous eosin for 2 minutes. Stained sections were dehydrated, placed in xylene, and mounted.

### Immunohistochemistry.

Immunohistochemistry on paraffin or cryosections was performed using standard methods (68, 69). Antibodies were as follows: guinea pig anti-PRDM13 (1:1000; described in ref. 33), rabbit anti–PC protein 2 (anti-PCP2; ref. 70) (1:200; gift from Brad Denker, Harvard University, Boston, Massachusetts, USA), mouse anti-LHX1,5 (1:100; Hybridoma Bank, catalog 4F2), rabbit anti-PAX2 (1:200; Thermo Fisher, catalog 71-600); guinea pig anti-TLX3 (71) (1:10000; gift from Thomas Muller, MDC Molecular Medicine, Berlin, Germany), anti-CC3 (Asp 175) (1:150; Cell Signaling Technology, catalog 9661), anti-phosphohistone H3B (Ser 10) (1:200; Abcam, catalog Ab14955), rabbit anti-GnRH (1:400; ImmunoStar, catalog 20075), rabbit anti-SOX9 (49) (1:1000; gift from Michael Wegner, FAU Institut für Biochemie, Erlangen-Nurnberg, Germany), rabbit anti-neurogranin (1:100; Merck Millipore, catalog AB5620), and rabbit anti-parvalbumin (1:200; Abcam, catalog AB11427). Sections were incubated with Alexa Fluor–labeled secondary antibodies (1:200; Life Technologies) and counterstained with DAPI to allow detection of PRDM13, PCP2, PAX2, TLX3, neurogranin, parvalbumin, CC3, and PH3B. Fluorescent images were acquired from Citifluor or Mowiol (Sigma-Aldrich) mounted slides using a Nikon Eclipse 80i microscope with a Nikon Y-QT Hamamatsu C4742-95 camera or with a Nikon A1R confocal microscope. For PAX2, TLX3, CC3, GnRH, and SOX9 detection, sections were incubated with species-specific biotinylated secondary antibody (1:200; Dako) for 1 to 2 hours. A VECTASTAIN Avidin/Biotin Complex (ABC) Kit (1:200; Vector Laboratories Ltd.) was used to amplify the signal prior to visualization. Sections were incubated in 3-3′-diaminobenzidine substrate (0.03%; Sigma-Aldrich), dehydrated where needed, mounted, and visualized with Nikon Eclipse 80i or Zeiss Axiovert microscopes. For detection of TLX3, cryosections were processed for antigen retrieval using 0.2% Triton X-100/PBS at 40°C for 20 minutes.

### In situ hybridization.

In situ hybridization on mouse sections was performed using previously described methods (72). DNA templates were generated from mouse genomic DNA by PCR amplification. The PCR primers used were as follows: *Prdm13* (exon4), forward, 5′-GCCACTTGTGCCTCTACTGT-3′; reverse, 5′-CCTCCACAGACAAGAGCGTT-3′. The T7 promoter was added at the 5′ end of the reverse primer. A digoxigenin (DIG) RNA-labeling kit with T7 RNA polymerase (Roche) was utilized to produce a *Prdm13* antisense probe by in vitro transcription. In situ hybridization on human tissue sections at CS23 of human embryonic development was carried out as previously described (73). The sections were prepared by the Human Developmental Biology Resource (HDBR), and a purified vector containing a conserved portion of human WT *PRDM13* cDNA (Source Bioscience) was used to make the DIG-labeled (Roche) sense and antisense RNA probes.

### RT-PCR and qRT-PCR.

Total RNA was extracted from samples (3 of each genotype) using TRIzol (Life Technologies) and Direct-zol RNA Miniprep Kit (Zymo Research). cDNA was synthesized from 200 ng of total RNA using the nanoScript2 Reverse Transcription Kit (Primer Design Ltd.) and random hexamer primers. Conventional PCR and sample analysis by gel electrophoresis were performed as previously described (74). qPCR was performed on a Roche LightCycler 480 qPCR machine using precision qPCR Master Mix with SYBR Green (PrimerDesign Ltd.). Triplicate samples were run in all reactions; first-strand DNA synthesis reactions without reverse transcriptase were used as controls. The quantification cycle (ΔCq) value and the ΔΔCq were calculated relative to control samples using Cq threshold values that were normalized to the housekeeping gene *Gapdh*.

Primer sequences are listed in Supplemental Methods, including those for the following: *PAX2*, *TLX3*, CC3, phosphohistone H3, parvalbumin, and neurogranin. Counts were performed on IHC-labeled cells on serial sagittal sections. The total numbers of labeled cells were counted using ImageJ (NIH) on images obtained using a Zeiss ApoTome microscope.

### GnRH neuron counts.

Counts were performed as previously described (69, 75).

### Area analysis.

The surface area of Cresyl violet (0.1%) serial sagittal sections was measured using ImageJ and used to estimate the cerebellar area.

### Molecular layer thickness.

Molecular layer thickness measurements were performed in the lobule III/IV region using PCP2-labeled sections, as previously described (50).

### Golgi-Cox staining.

Golgi-Cox staining was performed with the FD Rapid Golgi Stain Kit (FD Neurotechnologies, PK401) on adult brain samples, according to the manufacturer’s instructions. Following staining, sections were dehydrated, cleared with xylene, and mounted. Images were obtained using a Leica DMI6000 microscope.

### Behavior: motor coordination and learning.

Motor coordination and learning were assessed from 42 days of age on an accelerating rotarod (Panlab Harvard Apparatus) as described previously (76).

### Puberty assessment.

*Prdm13+/+* and *Prdm13–/–* female mice were checked daily for vaginal opening as previously described (77).

### Data availability.

Data were deposited in the European Genome-Phenome Archive (EGAS00001005878).

### Statistics.

Statistical tests employed are outlined in figure legends and were conducted when the experiment had been performed a minimum of 3 times on a minimum of 3 individual samples. Data are presented as mean ± SEM and results considered significant with a *P* value of less than 0.05.

### Study approval.

Animal housing and experimental procedures complied with the local ethical review panel of King’s College London, the United Kingdom Home Office Animals Scientific Procedures Act 1986, and Italian law (D. Lgs n° 2014/26, implementation of the 2010/63/UE). The work was performed under project licenses (PPL70/6694, PPL70/7184, and P8DC5B496 to MAB) and was approved by the University of Milan Animal Welfare Body and by the Italian Minister of Health (to AC). The appropriate ethical approval for the genetics and human embryonic tissue expression studies was obtained prior to this project taking place. Human embryonic and fetal material was provided by the Joint MRC/Wellcome Trust (grant no. MR/R006237/1; tissue sections obtained from HDBR, http://hdbr.org). Ethical committee approval for study of patient DNA samples was obtained from the Institute of Child Health/Great Ormond Street Hospital (GOSH) for Children Joint Research Ethics Committee. Informed consent was obtained from the parents of the patients prior to collection of samples and genomic analysis.

## Author contributions

The research was performed in 3 laboratories (PIs: MAB, AC, MTD). DEW characterized the cerebellar phenotypes in Prdm13-deficient mice, maintained the mouse colony, and collected all mouse in vivo data and tissues. RO investigated the hypothalamo/pituitary/gonadal axis in these mice. LCG performed the human genetic studies in collaboration with GOSGene and performed a detailed description of the phenotypes. She also performed in situ hybridization on human embryonic sections. While the contributions of all three first authors were critical to understanding the role of PRDM13 in hypothalamic and cerebellar development, the work on the cerebellar phenotype was complex and particularly difficult to perform during the COVID-19 lockdown and merited DEW the first place in the order of the first authors. Similarly, the murine work performed on the hypothalamo/pituitary/gonadal axis also took a considerable amount of time, particularly during the COVID-19 lockdown period, and merited RO second place on the shared first authorship. While LCG’s contribution was considerable and significant, it was felt that the collaboration with GOSGene meant that the work on the identification of the mutation was shared with GOSGene and so she would be third on the list of authors, while acknowledging that the manuscript could not have been published without the insights from the human genetics data. All three first authors agreed to this order of first authorship. MTD, HJW, MAB, and AC conceived the project and coordinated and supervised experimental work. LCG performed the human expression studies and generated the table of clinical patient data and the pedigree in Figure 1A. JGT, NF, MJC, and MTD recruited the patients to the study and performed phenotypic characterization of the patients. Colleagues from GOSgene HJW, PLQS, and LAO performed the next-generation sequencing analysis, variant interpretation, and Sanger sequencing of all participants. HJW analyzed the genome-wide microarray data. TC and TF provided *Prdm13*-deficient and control tissues and animals and PRDM13-specific antiserum. DEW, KLHR, and MAB established and maintained the *Prdm13* mouse colony at King’s College London. DEW, DF, SHR, and KLHR performed the analysis of cerebellar development. RO, AL, LBDM, and AJJP performed the analysis related to the GnRH and Kiss1 neuronal systems, including the histological analyses of gonads. RO and AC prepared Figures 1–4 and Supplemental Figure 2. DEW, RO, LCG, ICAFR, AC, MAB, and MTD wrote the manuscript with contributions from all authors.

## Supplementary Material

Supplemental data

## Version 1 11/02/2021

In-Press Preview

## Version 2 12/15/2021

Electronic publication
