# Supplementary material for: A recessive PRDM13 mutation results in congenital hypogonadotropic hypogonadism and cerebellar hypoplasia
Source: J Clin Invest. 2021 Dec 15;131(24):e141587. doi: 10.1172/JCI141587 (PMC8670848; doi:10.1172/JCI141587)
Supplement: Supplemental data [file jci-131-141587-s152.pdf]

Supplemental information

Supplemental Table 1: Summary of patient phenotypes

Patient	Age at Testing (years)	Karyotype	Birth weight Kg (SDS)	GnRH test (IU/L)	Time (mins)			hCG test: Serum Free Testosterone (nmol/L)	Scan: MRI or CT	Other features (age in years)	Treatment (age started in years)
					0	+20	+60				
1	14.3	XY	2.8<br>(-1.85)	LH	0.1	2.3	2.3	Baseline: 2.3<br><br>After 3 day hCG (day 4): 2.2	CT: cerebellar hypoplasia - hemispheres and vermis	Delayed motor development (1)<br>BL orchidopexy (3), Repeated left-side (6)<br>Right-sided thoracic progressive scoliosis (1.5): 2-stage surgical fixation of the spine (10)<br>Hypotonia<br>Symmetrical hypo-reflexia<br>Ataxic gait<br>Moderate intellectual deficit	Testosterone (14.5)
				FSH	0.2	2.6	4.4				
2	12.5	XX	3.4<br>(-0.11)	LH	<0.1	2.4	2.6	N/A	MRI: Normal	Generalised hypotonia<br>Hyporeflexia<br>Delayed gross motor development<br>Right-sided thoraco-lumbar progressive scoliosis (2): surgical fixation of the spine (16)<br>Moderate intellectual deficit	Ethinyloestradiol (13)<br>Norethisterone (15)
				FSH	0.8	3.1	6.4				
3	13	XY	2.95<br>(-1.47)	LH	<0.1	1.0	0.9	Baseline: <0.69<br><br>After 3 day hCG (day 4): 2.2<br><br>After 3 weeks hCG (day 20): 30.7	CT: cerebellar hypoplasia	Global developmental delay<br>Generalised hypertonia and hyper-reflexia (0.25)<br>Strabismus<br>Scoliosis: corrective surgery (11)<br>Wheelchair-dependent (12)<br>BL orchidopexy (14)<br>Severe intellectual deficit<br>Epilepsy (16)	Testosterone (13.5)<br>Lamotrigine (16)
				FSH	0.4	1.7	3.1				

Patients clinical data:

Pedigree I

Patient 1 (VI.3) is a Maltese patient born to a consanguineous union with a birth weight of 2.8 kg (-1.85 SDS). He was diagnosed with delayed motor development at 1 year of age, and noted to have a right-sided thoracic scoliosis from 18 months age. Neurological examination at 3 years revealed hypotonia, symmetrical hypo-reflexia, and an ataxic gait. He had bilateral undescended testes and underwent bilateral orchidopexy, which was repeated on the left at 6 years of age. MRI of the brain revealed hypoplasia of the cerebellar hemispheres and vermis (Figure 1B). Progression of scoliosis necessitated a 2-stage surgical fixation of the spine at the age of 10 years. He was referred to the Paediatric Endocrine Clinic at 14.3 years with delayed puberty. Pubertal staging at this age was G1 P1 A1 -/03 mL, and his height was 136 cm (-3.22 SDS). He had a healthy 18 year old sister (VI.2), and a 2 year old sister with neurological problems who was eventually diagnosed to have HH (Patient VI.4). A gonadotropin-releasing hormone (GnRH) test revealed a peak LH of 2.3 IU/L, with an FSH of 4.4 IU/L. A 3-day hCG test revealed no change in the testosterone concentration after 3 hCG injections (peak testosterone of 2.2 nmol/L), and was therefore suboptimal and consistent with hypogonadotrophic hypogonadism (Segal TY et al J Clin Endocrinol Metab. 2009 Mar;94(3):780-5). All other pituitary function tests (including thyroid function tests) were normal. Treatment with exogenous intramuscular testosterone (testosterone esters) was commenced at 14.5 years of age with a gradual increase in dosage over 2 years. Treatment was switched to a transdermal patch preparation at 17 years of age, and to long-acting testosterone undecanoate, by intramuscular injection every 3 months, at 22 years. He is now 30 years old with moderate intellectual deficit, and remains on testosterone replacement therapy.

Patient 2 (VI.4), the sister of Patient 1, had a birth weight of 3.4 kg (-0.11 SDS). At postnatal follow-up, she was noted to have generalised hypotonia and hyporeflexia, as well as delayed gross motor development. A progressive right-sided thoraco-lumbar scoliosis was first noted at the age of 2 years. All neurological investigations, including metabolic screen, EMG, and brain MRI were reported as normal. She was first seen in the Paediatric Endocrinology Clinic at the age of 11.3 years, with a similar neurological condition to her elder brother (Patient 1). She had not entered spontaneous puberty by the age of 12.5 years, with low basal gonadotrophins (basal LH <0.1 U/L, FSH 0.8 IU/L), and with a peak LH of 2.6 IU/L and an FSH of 6.4 IU/L on GnRH testing.

All other pituitary function tests (including thyroid function and serum prolactin) were normal. At 13 years of age, when her pubertal staging remained at Tanner B1, she was started on oral ethinyloestradiol to induce pubertal development. The dose of ethinyloestradiol was increased gradually over the subsequent 2 years, which resulted in normal breast development (Tanner B4 by 15 years of age). Oral norethisterone was introduced at the age of 15 years in order to induce regular menstrual periods. Surgical fixation of the spine was recently performed at 16 years of age. She is now 16.5 years old with moderate intellectual deficit, and remains on oral ethinyloestradiol and norethisterone.

<u>Pedigree II</u>

Patient 3 was born to healthy, non-consanguineous Maltese parents, with a birth weight of 2.95 kg (-1.47 SDS). The pregnancy was complicated by hyperemesis gravidarum and polyhydramnios, with reported intrauterine growth restriction. Global developmental delay, generalised hypertonia and hyper-reflexia were first noted at 3 months of age. He also had bilateral epicanthic folds and downturned angles of the mouth. A CT brain scan revealed cerebellar hypoplasia. Initially, he was able to walk with a very broad-based gait using a walking frame with significant support. He needed corrective surgery for strabismus as well as spinal surgery for progressive scoliosis at 11 years of age, but became completely wheelchair-dependent by the age of 12 years. He was referred to the Paediatric Endocrine Clinic at the age of 11.4 years with a micropenis. On pubertal staging at this age, his stretched penile length was 4 cm (less than P10) and both testes were impalpable. Basal gonadotrophins were low (LH <0.1 IU/L, FSH 0.4 IU/L). A GnRH test performed at the age of 13 years revealed a peak LH of 0.9 IU/L with an FSH of 3.1 IU/L. The peak testosterone was sub-optimal at 2.3 nmol/L after a 3 day hCG test, with an excellent peak of 30.7 nmol/L after 3 weeks of HCG. Following these tests, the left testis descended into the scrotum (2 mL volume), but the right testis remained impalpable. Exogenous testosterone (testosterone enantate) was commenced at low dose by intramuscular injection at 13.5 years of age, and the dose increased gradually over the following 2 years. Bilateral orchidopexies were performed at 14 years of age. Over time, he experienced penile growth, but both testes remained 2 mL in volume. He is currently 21 years old with severe intellectual deficit, and continues to receive exogenous testosterone replacement therapy. He also began treatment with oral lamotrigine for epilepsy at the age of 16 years. He has a healthy brother who is 2 years older, and who had gone through puberty spontaneously at 12 – 13 years of age.

Supplemental Table 2: Neurological phenotype of affected patients

**Information**		**Patient 1 (VI.3)**	**Patient 2 (VI.4)**	**Patient 3 (Pedigree 2)**
Neurological information	Tone	Generalised hypotonia	Generalised hypotonia	Generalised hypotonia
	Power	Reduced	Very reduced	Extremely reduced
	Reflexes	Symmetrical hyporeflexia<br>Down-going plantar reflexes	Symmetrical hyporeflexia<br>Down-going plantar reflexes	Symmetrical hyporeflexia<br>Down-going plantar reflexes
	Gait	Broad-based, but able to ambulate by using a frame	Broad-based, but able to ambulate by using a frame	Initially very broad-based, later completely wheelchair-dependent
	Nystagmus	Not present	Not present	Present
	Intention tremor	Present	Present	Present
	Dysdiadochokinesis	Present	Present	Present
	Past-pointing	Present	Present	Present
Age measured		28 years	17 years	17 years
Weight		62 kg	56 kg	36.4 kg
Height		155.4 cm	144.5 cm	Impossible
BMI		25.6 kg/m^2	26.8 kg/m^2	?

Supplemental Table 3: A region on chromosome 6 surrounding the *PRDM13* mutation (c.398-3_407delCAGGGGAGGAGCG), showing the long-affected only haplotype (chr6:98896667-chr6:100480906=1584239bp/1.6Mb) and the nested-shared haplotype (chr6:100053626-chr6:100265121=211495bp/0.2Mb). Genotype data was generated from Illumina Infinium OmniExpress-48 microarray, all genomic positions correspond to genome build GRCh37/Hg19.

			Patient 1	Patient 2	Patient 3	Control	
Name	Chr	Position	Hom	Hom	Hom	Het Carrier	
rs3104095	6	98883438	AA	AA	AB	BB	
rs1494777	6	98892120	AA	AA	AB	AB	
rs3123339	6	98893182	BB	BB	AB	AB	
rs3125575	6	98895069	AA	AA	AB	AB	
rs9321063	6	98896667	AA	AA	AA	AA	Beginning of long-affected only haplotype
rs17761570	6	98904572	AB	AB	AB	AB	
rs9388482	6	98905933	BB	BB	BB	AB	
rs9491633	6	98914680	BB	BB	BB	BB	
rs9491646	6	98927363	BB	BB	BB	BB	
rs210400	6	98931278	AA	AA	AA	AA	
rs169750	6	98931716	BB	BB	BB	BB	
rs9398813	6	98935310	BB	BB	BB	BB	
rs9321067	6	98936578	BB	BB	BB	BB	
rs17058578	6	98938023	BB	BB	BB	BB	
rs9401901	6	98938237	AA	AA	AA	AB	
rs9388508	6	98938249	BB	BB	BB	BB	
rs2227121	6	98938834	AB	AB	BB	AB	
rs158773	6	98949550	BB	BB	BB	BB	
rs158774	6	98953633	AA	AA	AA	AB	
rs158777	6	98959605	AA	AA	AA	AB	
rs150396	6	98962591	BB	BB	BB	BB	

rs1481449	6	98971740	BB	BB	BB	BB	
rs211215	6	98975810	AA	AA	AA	AA	
rs11961327	6	98978496	BB	BB	BB	BB	
rs211221	6	98980113	BB	BB	BB	BB	
rs211222	6	98980147	BB	BB	BB	AB	
rs17058667	6	98991464	BB	BB	BB	BB	
rs183316	6	98992369	BB	BB	BB	BB	
rs211227	6	99001499	AA	AA	AA	AA	
rs2046174	6	99013740	AA	AA	AA	AA	
rs17829357	6	99017732	BB	BB	BB	BB	
rs9491794	6	99019431	BB	BB	BB	AB	
rs9401968	6	99027074	BB	BB	BB	BB	
rs1481440	6	99032723	AA	AA	AA	AB	
rs11752460	6	99033489	AA	AA	AA	AA	
rs1481438	6	99034313	AA	AA	AA	AB	
rs6929790	6	99045749	AA	AA	AA	AB	
rs17830067	6	99050825	BB	BB	BB	BB	
rs969540	6	99058170	BB	BB	BB	BB	
rs17058737	6	99076746	AA	AA	AA	AA	
rs11757142	6	99080658	AA	AA	AA	AA	
rs17058761	6	99092539	BB	BB	BB	BB	
rs11752997	6	99124184	AA	AA	AA	AA	
rs4472356	6	99126082	AA	AA	AA	AB	
rs9375573	6	99126994	AA	AA	AA	AB	
rs12204275	6	99138875	BB	BB	BB	BB	
rs6939572	6	99139350	AA	AA	AA	AA	
rs6924761	6	99140492	BB	BB	BB	BB	
rs4424090	6	99147074	BB	BB	BB	BB	
rs9375604	6	99170039	AA	AA	AA	AA	
rs4529305	6	99173425	AA	AA	AA	AA	
rs9388676	6	99175625	BB	BB	BB	BB	

rs11154467	6	99195548	BB	BB	BB	AB	
rs4406241	6	99203222	BB	BB	BB	BB	
rs7762570	6	99214884	AA	AA	AA	AB	
rs4839976	6	99218958	AA	AA	AA	AB	
rs4398748	6	99219522	AA	AA	AA	AA	
rs9385493	6	99221669	AA	AA	AA	AB	
rs10499023	6	99226729	AA	AA	AA	AA	
rs4839977	6	99229574	BB	BB	BB	AB	
rs4555920	6	99230880	BB	BB	BB	AB	
rs3923049	6	99233448	AA	AA	AA	AB	
rs9375642	6	99235909	AA	AA	AA	AB	
rs9402154	6	99236649	BB	BB	BB	AB	
rs9388719	6	99237315	BB	BB	BB	AA	
rs12174549	6	99237479	AA	AA	AA	AA	
rs7762652	6	99242592	BB	BB	BB	AA	
rs9385517	6	99250916	BB	BB	BB	BB	
rs4839986	6	99251754	BB	BB	BB	AB	
rs6569636	6	99258445	AA	AA	AA	BB	
rs9375688	6	99270438	BB	BB	BB	AB	
rs9375689	6	99270533	BB	BB	BB	AA	
rs2444935	6	99275290	BB	BB	BB	BB	
rs1869641	6	99277867	AA	AA	AA	BB	
rs1883306	6	99279449	AA	AA	AA	AB	
rs3823036	6	99284532	AA	AA	AA	AB	
rs195853	6	99290334	BB	BB	BB	BB	
rs195852	6	99290592	AA	AA	AA	BB	
rs195851	6	99294322	BB	BB	BB	AA	
rs174447	6	99309057	AA	AA	AA	BB	
rs9388789	6	99320715	BB	BB	BB	AB	
rs11537982	6	99323424	BB	BB	BB	BB	
rs9375728	6	99326490	AA	AA	AA	AB	

rs195831	6	99328730	AA	AA	AA	AA	
rs10484609	6	99336279	AA	AA	AA	AB	
rs7739884	6	99346145	BB	BB	BB	AB	
rs195824	6	99351047	AA	AA	AA	AB	
rs1011676	6	99374400	BB	BB	BB	BB	
rs17058986	6	99431200	AA	AA	AA	AA	
rs196959	6	99432747	BB	BB	BB	BB	
rs196960	6	99433614	BB	BB	BB	AA	
rs2747734	6	99437566	BB	BB	BB	AA	
rs2572109	6	99440369	BB	BB	BB	AB	
rs12200990	6	99441083	BB	BB	BB	AB	
rs2180046	6	99450081	BB	BB	BB	BB	
rs2747739	6	99462075	BB	BB	BB	AB	
rs9402354	6	99462324	AA	AA	AA	AA	
rs9375844	6	99463771	BB	BB	BB	BB	
rs2207446	6	99464542	AA	AA	AA	BB	
rs12173555	6	99467661	BB	BB	BB	AB	
rs9388950	6	99468942	AA	AA	AA	BB	
rs2092772	6	99473410	BB	BB	BB	AB	
rs12201236	6	99478174	BB	BB	BB	AB	
rs4839737	6	99478304	AA	AA	AA	BB	
rs2747748	6	99479842	AA	AA	AA	AA	
rs7756447	6	99480633	BB	BB	BB	BB	
rs11756151	6	99485266	BB	BB	BB	BB	
rs1997937	6	99487230	BB	BB	BB	AB	
rs12173832	6	99498599	BB	BB	BB	BB	
rs2144241	6	99500963	AA	AA	AA	AA	
rs6916751	6	99501709	AA	AA	AA	AA	
rs9493282	6	99504377	BB	BB	BB	BB	
rs12660289	6	99515134	AA	AA	AA	AB	
rs2207445	6	99521031	BB	BB	BB	BB	

rs7764372	6	99524062	BB	BB	BB	BB	
rs9375894	6	99524281	AA	AA	AA	AB	
rs12212298	6	99531172	AA	AA	AA	AA	
rs9385615	6	99531774	BB	BB	BB	AB	
rs2572098	6	99538132	AA	AA	AA	AA	
rs9402444	6	99539153	BB	BB	BB	AB	
rs6902772	6	99550464	BB	BB	BB	AB	
rs6940049	6	99551169	AA	AA	AA	AA	
rs3860236	6	99551765	BB	BB	BB	AB	
rs10457592	6	99551970	BB	BB	BB	BB	
rs12208335	6	99552868	AA	AA	AA	AB	
rs6938641	6	99557664	AA	AA	AA	AA	
rs9373032	6	99559165	BB	BB	BB	AB	
rs1081025	6	99559773	AA	AA	AA	AB	
rs12526079	6	99565215	BB	BB	BB	BB	
rs7769941	6	99569709	AA	AA	AA	AA	
rs2388839	6	99574363	BB	BB	BB	AB	
rs12111251	6	99575661	BB	BB	BB	BB	
rs4454147	6	99575874	BB	BB	BB	BB	
rs2029964	6	99583773	AA	AA	AA	AB	
rs728758	6	99584658	BB	BB	BB	BB	
rs11154718	6	99592404	BB	BB	BB	AB	
rs12190591	6	99593258	AA	AA	AA	AA	
rs9321394	6	99600083	AA	AA	AA	AB	
rs11754157	6	99607499	BB	BB	BB	BB	
rs4839999	6	99610074	BB	BB	BB	AB	
rs12206927	6	99613136	BB	BB	BB	AA	
rs12110525	6	99629252	BB	BB	BB	BB	
rs9373057	6	99630038	BB	BB	BB	AB	
rs9399068	6	99633011	AA	AA	AA	BB	
rs9375977	6	99637123	BB	BB	BB	AA	

rs7745052	6	99640610	BB	BB	BB	AB		
rs1496971	6	99641248	AA	AA	AA	BB		
rs1908804	6	99646240	AA	AA	AA	AA		
rs7767885	6	99659802	AA	AA	AA	BB		
rs7769752	6	99662681	AA	AA	AA	BB		
rs17059246	6	99662743	AA	AA	AA	AB		
rs9389116	6	99664294	BB	BB	BB	AB		
rs4458696	6	99665178	BB	BB	BB	BB		
rs9373072	6	99669676	BB	BB	BB	AB		
rs2132683	6	99672014	AA	AA	AA	AB		
rs9375997	6	99673152	BB	BB	BB	AB		
rs9402556	6	99673466	AA	AA	AA	BB		
rs12528619	6	99675908	BB	BB	BB	BB		
rs1566116	6	99678424	AA	AA	AA	AA		
rs4840017	6	99679578	BB	BB	BB	BB		
rs9402564	6	99679902	BB	BB	BB	BB		
rs10155713	6	99683181	BB	BB	BB	BB		
rs2029965	6	99683802	BB	BB	BB	BB		
rs9376014	6	99690090	AA	AA	AA	AA		
rs12193060	6	99690449	AA	AA	AA	AA		
rs1874538	6	99694494	AA	AA	AA	AA		
rs6904604	6	99695108	BB	BB	BB	BB		
rs9493928	6	99711169	BB	BB	BB	BB		
rs9483707	6	99717329	BB	BB	BB	AB		
rs13219146	6	99717914	AA	AA	AA	AB		
rs1045728	6	99721049	BB	BB	BB	AB		
rs12660321	6	99727653	BB	BB	BB	BB		
rs1496979	6	99728442	AA	AA	AA	AB		
rs6933093	6	99729901	BB	BB	BB	AB		
rs9373105	6	99733939	AA	AA	AA	AA		
rs1496980	6	99734185	BB	BB	BB	AB		

rs12207550	6	99740492	BB	BB	BB	BB	
rs221582	6	99744107	BB	BB	BB	AB	
rs221578	6	99747633	AA	AA	AA	AA	
rs221530	6	99753499	AA	AA	AA	AA	
rs6913076	6	99763266	BB	BB	BB	BB	
rs182613	6	99768894	BB	BB	BB	AB	
rs221527	6	99771540	BB	BB	BB	BB	
rs13206094	6	99772374	AA	AA	AA	AB	
rs4840031	6	99773417	BB	BB	BB	BB	
rs6922449	6	99776986	AA	AA	AA	AB	
rs11757364	6	99781218	BB	BB	BB	AB	
rs17059400	6	99782879	AA	AA	AA	AA	
rs11963108	6	99793615	BB	BB	BB	BB	
rs9402701	6	99801698	BB	BB	BB	AB	
rs12198238	6	99803269	BB	BB	BB	AB	
rs13194648	6	99804157	BB	BB	BB	AB	
rs17059457	6	99806408	AA	AA	AA	AA	
rs4839747	6	99814530	BB	BB	BB	BB	
rs6925344	6	99819379	BB	BB	BB	AB	
rs12193590	6	99826265	BB	BB	BB	AB	
rs9402716	6	99827314	BB	BB	BB	AB	
rs4840038	6	99828941	AA	AA	AA	AB	
rs11154812	6	99836954	BB	BB	BB	BB	
rs9376137	6	99842056	BB	BB	BB	BB	
rs9376138	6	99842138	BB	BB	BB	BB	
rs11961608	6	99847168	AA	AA	AA	AA	
rs4144165	6	99847260	BB	BB	BB	BB	
rs3811072	6	99851977	BB	BB	BB	BB	
rs11154824	6	99852267	AA	AA	AA	AA	
rs4840039	6	99869689	BB	BB	BB	BB	
rs12198321	6	99871010	BB	BB	BB	BB	

rs4351270	6	99873534	BB	BB	BB	BB	
rs6923983	6	99877219	AA	AA	AA	AB	
rs1134718	6	99880380	AA	AA	AA	AA	
rs12214037	6	99880572	AA	AA	AA	AA	
rs17785525	6	99882563	BB	BB	BB	AB	
rs6570064	6	99883137	AA	AA	AA	AB	
rs9402791	6	99883694	AA	AA	AA	AA	
rs6570065	6	99883704	BB	BB	BB	BB	
rs4839748	6	99889915	AA	AA	AA	AB	
rs9494471	6	99893527	BB	BB	BB	AB	
rs12203426	6	99893878	AA	AA	AA	AA	
rs4504482	6	99893938	AA	AA	AA	AA	
rs10155760	6	99901608	BB	BB	BB	BB	
rs6916603	6	99905558	BB	BB	BB	AB	
rs10457650	6	99916182	AA	AA	AA	AA	
rs7745012	6	99921822	BB	BB	BB	BB	
rs9483935	6	99933680	BB	BB	BB	BB	
rs6918880	6	99934446	AA	AA	AA	AA	
rs12717185	6	99939045	AA	AA	AA	AB	
rs2209157	6	99964012	BB	BB	BB	BB	
rs9402863	6	99971429	BB	BB	BB	AB	
rs10223892	6	99973893	BB	BB	BB	AB	
rs17224695	6	99974948	BB	BB	BB	AB	
rs2057517	6	99980252	AA	AA	AA	AA	
rs2057518	6	99980522	AA	AA	AA	AA	
rs7754710	6	99986195	BB	BB	BB	AB	
rs1054227	6	99990856	BB	BB	BB	AB	
rs2296154	6	99993268	AA	AA	AA	AB	
rs13205324	6	99995320	BB	BB	BB	BB	
rs543967	6	100005775	BB	BB	BB	AB	
rs1590359	6	100022125	BB	BB	BB	BB	

rs514769	6	100031037	AA	AA	AA	AB	
rs1552855	6	100046845	AA	AA	AA	AB	
rs9484083	6	100046933	AA	AA	AA	AB	
rs7741279	6	100053626	BB	BB	BB	BB	Beginning of nested-shared haplotype
rs330843	6	100060761	BB	BB	BB	BB	
	**6**	**100060906**					**Mutation position**
rs3734346	6	100062766	AA	AA	AA	AA	
rs330844	6	100063243	BB	BB	BB	BB	
rs6927488	6	100081441	BB	BB	BB	BB	
rs594231	6	100082983	BB	BB	BB	BB	
rs9402954	6	100088902	AA	AA	AA	AA	
rs1339203	6	100092390	BB	BB	BB	BB	
rs546567	6	100099703	BB	BB	BB	BB	
rs13193313	6	100108500	AA	AA	AA	AA	
rs9321659	6	100116092	BB	BB	BB	BB	
rs650783	6	100125119	AA	AA	AA	AA	
rs9373217	6	100129570	BB	BB	BB	BB	
rs503649	6	100129959	AA	AA	AA	AA	
rs472977	6	100130991	BB	BB	BB	BB	
rs9495145	6	100131219	BB	BB	BB	BB	
rs9376355	6	100132920	AA	AA	AA	AA	
rs12661094	6	100161035	BB	BB	BB	BB	
rs12211649	6	100172277	BB	BB	BB	BB	
rs4839755	6	100173173	BB	BB	BB	BB	
rs9389645	6	100173832	AA	AA	AA	AA	
rs6916754	6	100174464	BB	BB	BB	BB	
rs6908196	6	100185261	BB	BB	BB	BB	
rs12527523	6	100187748	BB	BB	BB	BB	
rs12525414	6	100192215	AA	AA	AA	AA	
rs17826560	6	100195578	AA	AA	AA	AA	
rs7742890	6	100197285	AA	AA	AA	NC	

rs7356874	6	100197712	BB	BB	BB	BB	
rs10485226	6	100206681	AA	AA	AA	AA	
rs10485227	6	100207327	BB	BB	BB	BB	
rs4388294	6	100207838	AA	AA	AA	AA	
rs17059765	6	100208624	AA	AA	AA	AA	
rs4431442	6	100213515	BB	BB	BB	BB	
rs9403091	6	100228256	BB	BB	BB	AB	
rs9376435	6	100231618	BB	BB	BB	BB	
rs9385869	6	100233278	AA	AA	AA	AA	
rs9399284	6	100233870	AA	AA	AA	AA	
rs12663112	6	100236473	AA	AA	AA	AA	
rs4596491	6	100236866	BB	BB	BB	BB	
rs12198721	6	100237464	AA	AA	AA	AA	
rs6936455	6	100237880	BB	BB	BB	BB	
rs9403103	6	100240267	BB	BB	BB	BB	
rs6929006	6	100242158	BB	BB	BB	BB	
rs6929428	6	100242366	BB	BB	BB	BB	
rs17236659	6	100243033	AA	AA	AA	AA	
rs9376456	6	100243997	BB	BB	BB	BB	
rs6931227	6	100247048	AA	AA	AA	AA	
rs7763983	6	100249106	BB	BB	BB	BB	
rs17059858	6	100250740	AA	AA	AA	AA	
rs9484314	6	100251499	AA	AA	AA	AA	
rs17059867	6	100252886	BB	BB	BB	BB	
rs9373250	6	100255471	BB	BB	BB	BB	
rs12216503	6	100257066	BB	BB	BB	BB	
rs4495279	6	100257668	BB	BB	BB	BB	
rs6934621	6	100265121	BB	BB	BB	BB	End of nested-shared haplotype
rs7760502	6	100266123	AA	AA	AA	AB	
rs17059881	6	100268849	AA	AA	AA	AA	
rs13204333	6	100277840	BB	BB	BB	AB	

rs9403141	6	100279956	BB	BB	BB	BB	
rs9385894	6	100280032	BB	BB	BB	BB	
rs7453413	6	100281817	AA	AA	AA	AB	
rs3922542	6	100282075	BB	BB	BB	AB	
rs9321763	6	100286127	BB	BB	BB	BB	
rs4370372	6	100288317	AA	AA	AA	AA	
rs9389752	6	100289337	BB	BB	BB	BB	
rs9495653	6	100292091	AA	AA	AA	AA	
rs9495657	6	100293531	AA	AA	AA	AA	
rs4990843	6	100296024	BB	BB	BB	BB	
rs9403163	6	100297481	AA	AA	AA	AA	
rs12197810	6	100317146	AA	AA	AA	AA	
rs9403180	6	100319031	AA	AA	AA	AA	
rs2397663	6	100321669	AA	AA	AA	AA	
rs2397664	6	100321734	AA	AA	AA	AA	
rs9403186	6	100324464	AA	AA	AA	AA	
rs9495745	6	100326214	BB	BB	BB	BB	
rs4840097	6	100328198	BB	BB	BB	AB	
rs9389810	6	100329446	AA	AA	AA	AB	
rs2397678	6	100345238	AA	AA	AA	AB	
rs6902801	6	100345403	AA	AA	AA	AA	
rs7751620	6	100353268	AA	AA	AA	AB	
rs9403208	6	100354935	BB	BB	BB	BB	
rs4560657	6	100366635	AA	AA	AA	AB	
rs13206575	6	100369284	BB	BB	BB	AB	
rs4559096	6	100391872	BB	BB	BB	AB	
rs12215494	6	100392532	BB	BB	BB	AB	
rs13195863	6	100393761	AA	AA	AA	AB	
rs10499026	6	100397526	AA	AA	AA	AB	
rs4840106	6	100403514	BB	BB	BB	BB	
rs7758072	6	100411417	BB	BB	BB	BB	

rs7739904	6	100427015	BB	BB	BB	BB	
rs9496070	6	100428029	BB	BB	BB	BB	
rs13212643	6	100428313	AA	AA	AA	AA	
rs12203515	6	100430062	BB	BB	BB	BB	
rs9496085	6	100430803	BB	BB	BB	BB	
rs9389934	6	100431321	AA	AA	AA	AA	
rs2001456	6	100439009	BB	BB	BB	AB	
rs6925272	6	100442268	BB	BB	BB	AB	
rs9969034	6	100442554	BB	BB	BB	BB	
rs2397694	6	100445429	AA	AA	AA	AA	
rs9399386	6	100446900	BB	BB	BB	AB	
rs3763374	6	100448788	BB	BB	BB	BB	
rs9385975	6	100449301	BB	BB	BB	BB	
rs9389951	6	100457411	BB	BB	BB	AB	
rs9484578	6	100469529	AA	AA	AA	AA	
rs4240585	6	100480906	BB	BB	BB	AB	End of long-affected only haplotype
rs6925134	6	100482016	AB	AB	BB	BB	
rs12202476	6	100492824	BB	BB	BB	BB	
rs3890820	6	100494186	BB	BB	BB	BB	
rs6903499	6	100510685	BB	BB	BB	BB	
rs11155243	6	100514432	BB	BB	BB	BB	
rs6938191	6	100521649	AB	AB	BB	AB	

Primer sequences:

qPCR primer sequences:

Gene	Forward Primer	Reverse Primer
Prdm13 exon 2-3	GGATAGGGTTAATCCGGGCA	TGGAGTCGCAGTTGTAGGGA
Prdm13 exon 4	GAGATCGCCATGCACACACAG	AGTACAGCTTGCCACAGTAGAG
Kiss1	ATGATCTCAATGGCTTCTTGG	CCAGGCATTAACGAGTTCCT
Npvf	CAAGACACCCGCTGATTTGC	TCCTCTCCTCGTTCGCTTTC
Pomc	TGGGCGAGCTGATGACCT	GCCGACTGTGAAATCTGAAAGG
Agrp	CTTTGGCGGAGGTGCTAGAT	AGGACTCGTGCAGCCTTACAC
Npy	TGGCCAGATACTACTCCGCT	TCCTCTCCTCGTTCGCTTTC
Gad1	CTTCTTCAGGCTCTCCCGTG	CAGGAACAGGCTCGGTTCAG
Gapdh	AGGTCGGTGTGAACGGATTTG	TGTAGACCATGTAGTTGAGGTCA

RT-PCR primer sequences:

Gene	Forward Primer	Reverse Primer
Prdm13	GCCACTTGTGCCTCTACTGT	CCTCCACAGACAAGAGCGTT
Gapdh	TGGCATTGTGGAAGGGCTCATGAC	ATGCCAGTGAGCTTCCCGTTCAGC

Genotyping primer sequences:

Gene	Forward Primer	Reverse Primer
**Prdm13 - KO**	CACCTCAGTCTTTGCCTTCCTTGCAA	CTACAACTGCGACTCCAACGCATGAT
**Prdm13 - WT**	CACCTCAGTCTTTGCCTTCCTTGCAA	CAGAGAAAGAGTACCCTTGTGCCT

Supplemental Figures:

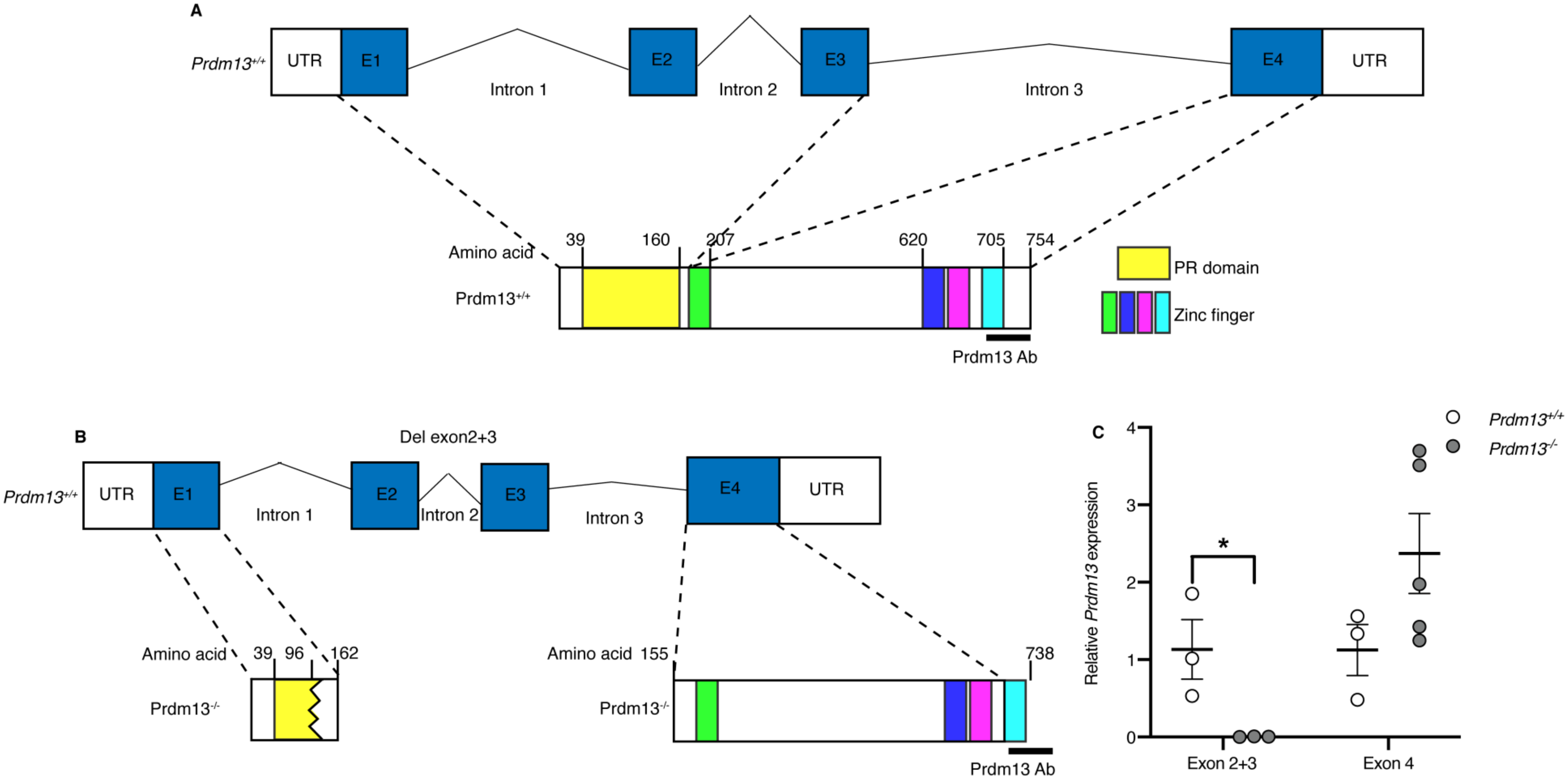

Supplemental Figure 1. *Prdm13*$^{\Delta ex2,3}$/$^{\Delta ex2,3}$ mutant allele. (A) Schematic representation of the mouse *Prdm13* locus and protein. The untranslated regions (UTR) are labelled. Exons are indicated by blue boxes and labelled E1, E2, E3 and E4. The functional domains encoded by the exons are shown and labelled according to the key provided. Dashed lines indicate the regions of the protein encoded by exon 1-3 and exon 4. (B) Schematic representation of the mouse *Prdm13* locus and the predicted *Prdm13*$^{-/-}$ protein. Predicted translated proteins are indicated and the C-terminal epitope of antiserum used to identify the wild-type protein is shown. (C) Quantification of *Prdm13* exon 2+3 and exon 4 transcript levels in E12.5 cerebella. Note the significant reduction in exon 2+3 expression in *Prdm13* mutants whilst exon 4 transcripts were increased but did not reach significance. *P<0.05, **P<0.01, two-tailed unpaired Student's *t* test. E1 = exon1, E2 = exon2, E3 = exon3, E4 = exon4, UTR = untranslated region.

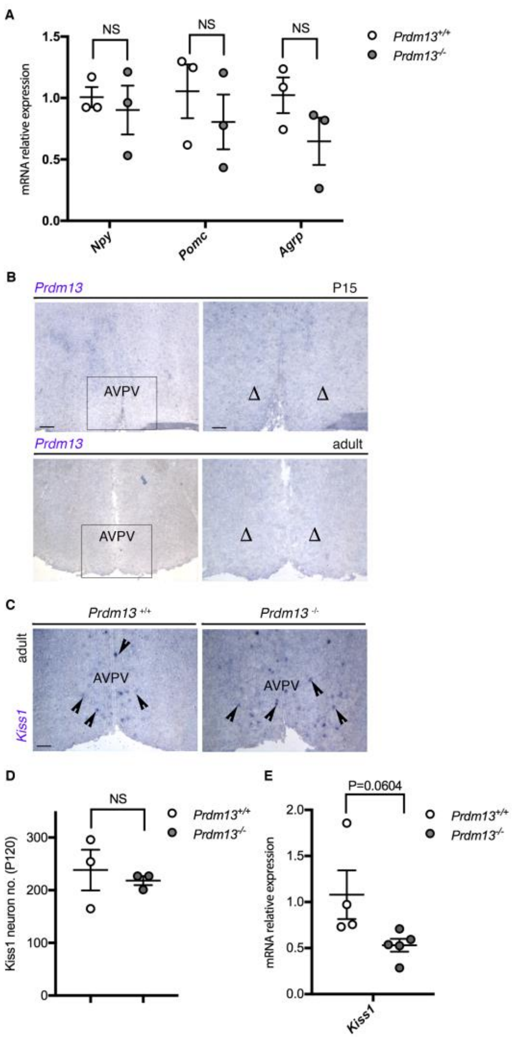

A
NS
NS
NS
Prdm13+/+
Prdm13-/-
mRNA relative expression
1.5
1.0
0.5
0.0
Npy
Pomc
Agrp
B
Prdm13
P15
AVPV
Δ Δ
Prdm13
adult
AVPV
Δ Δ
C
Prdm13 +/+
Prdm13 -/-
adult
Kiss1
AVPV
AVPV
D
NS
Prdm13+/+
Prdm13-/-
Kiss1 neuron no. (P120)
300
200
100
0
E
P=0.0604
Prdm13+/+
Prdm13-/-
mRNA relative expression
2.0
1.5
1.0
0.5
0.0
Kiss1

Supplemental Figure 2. *Prdm13*-null mice display similar levels of Arc GABAergic neuronal markers and a similar number of AVPV Kiss1 neurons compared to wild-type mice.

(A) qRT-PCR analysis for *Npy, Pomc, Agrp* transcripts in the hypothalamus of *Prdm13*$^{+/+}$ and *Prdm13*$^{-/-}$ male mice. ΔΔCq were calculated relative to control samples using quantification cycle (Cq) threshold values that were normalised to the housekeeping gene, *Gapdh*.
(B) *In situ* hybridization experiments on coronal P15 and adult male brain sections to detect the expression of *Prdm13* in the AVPV nucleus of the hypothalamus. High magnification of the squared areas are shown next to each panel. Note the absence of *Prdm13* transcript expression in the AVPV nucleus at both stages (Δ).
(C) *In situ* hybridization experiments on sections taken at the level of the AVPV nucleus from *Prdm13*$^{+/+}$ and *Prdm13*$^{-/-}$ adult male mice for *Kiss1* transcripts. Arrowheads indicate *Kiss1*-positive neurons.
(D) Quantification of Kiss1-positive neurons in the AVPV nucleus from *Prdm13*$^{+/+}$ and *Prdm13*$^{-/-}$ adult male mice. No differences in *Kiss1* expression were observed in the mutants compared to wild-types.
(E) qRT-PCR analysis for *Kiss1* transcript in the hypothalamus of *Prdm13*$^{+/+}$ and *Prdm13*$^{-/-}$ female mice. ΔΔCq were calculated relative to control samples using Cq threshold values that were normalised to the housekeeping gene, *Gapdh*. Note the non-significantly decreased *Kiss1* levels in mutant mice compared to wild-type.
Scale bars = 500 µm (B low magnification), 250 µm (B high magnification, C).
AVPV = Anteroventral periventricular nucleus.

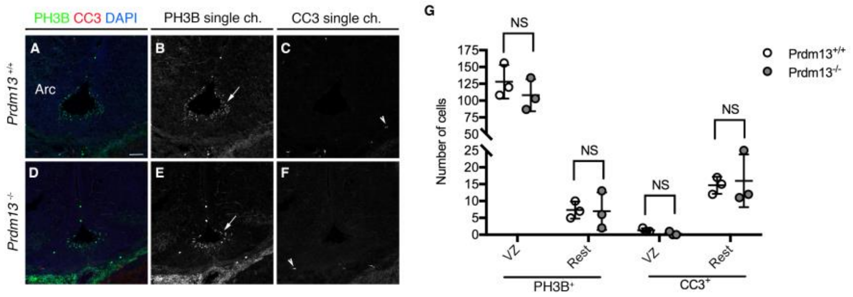

Supplemental figure 3. Loss of Kiss1 neurons in the developing hypothalamus of *Prdm13*$^{-/-}$ mice is not associated with altered cell proliferation or cell death.

(A-F) PH3B (green) and CC3 (red) co-immunostained representative sections from E14.5 embryos of indicated genotypes at the level of the developing Arc nucleus of the hypothalamus. Single channels are shown beside each image (B,C,E,F). Arrows indicate examples of PH3B^{+} cells; arrowheads indicate CC3^{+} cells.

(G) Quantification of proliferating PH3B^{+} and apoptotic CC3^{+} cells in the VZ and in the rest of the developing hypothalamus. No significant difference in proliferation was noted between wild-types and mutants.

Scale bar = 250µm.

Arc = arcuate nucleus, VZ = ventricular zone.

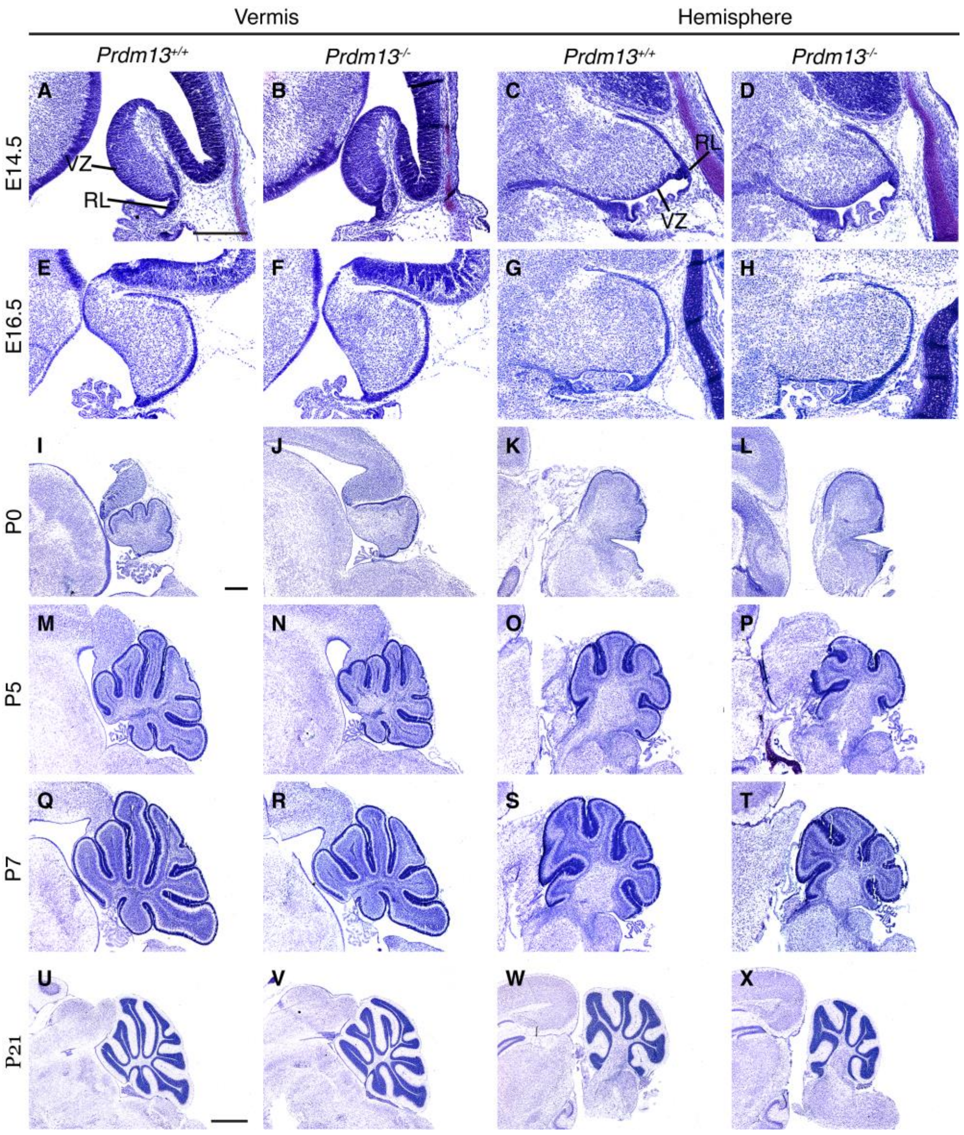

Vermis
Hemisphere
Prdm13+/+
Prdm13-/-
Prdm13+/+
Prdm13-/-
E14.5
E16.5
P0
P5
P7
P21
VZ
RL
RL
VZ
A
B
C
D
E
F
G
H
I
J
K
L
M
N
O
P
Q
R
S
T
U
V
W
X

Supplemental Figure 4. Time course of *Prdm13*$^{-/-}$ cerebellar development demonstrating cerebellar hypoplasia at early postnatal stages. Cresyl violet-stained sagittal sections through the cerebellar vermis and hemisphere of *Prdm13*$^{+/+}$ and *Prdm13*$^{-/-}$ mice at indicated stages (A-X). Note subtle abnormalities in cerebellar vermis foliation at P0 and overt cerebellar hypoplasia in the vermis by P5 and the hemispheres by P7. Note that images Q, R, W, X are also presented in Figure 5U, V, O, P, respectively. VZ = ventricular zone, RL = rhombic lip. Scale bar = 300µm (A,I), 1mm (U).

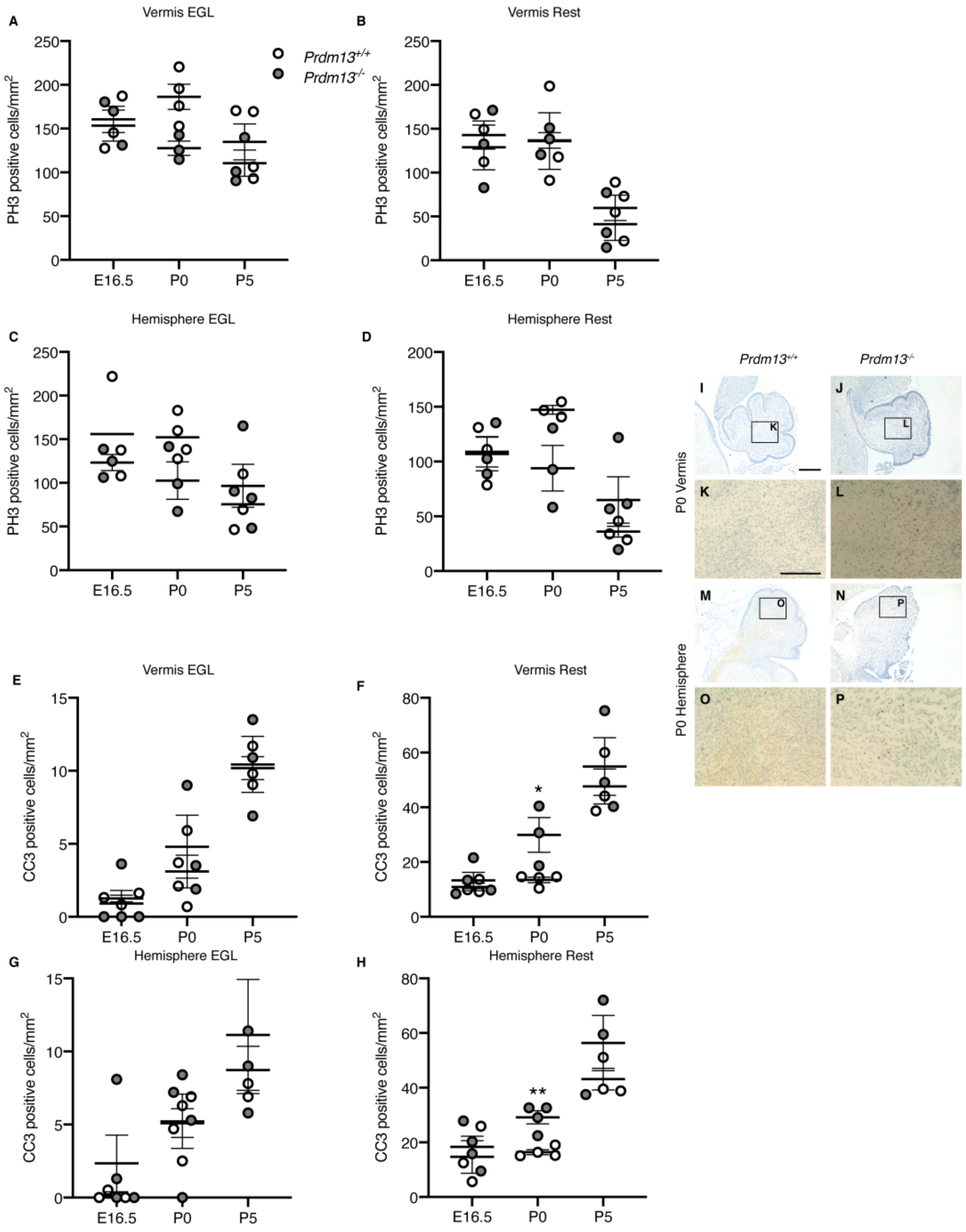
A
Vermis EGL
B
Vermis Rest
Prdm13+/+
Prdm13-/-
PH3 positive cells/mm2
E16.5
P0
P5
C
Hemisphere EGL
D
Hemisphere Rest
Prdm13+/+
Prdm13-/-
I
J
K
L
P0 Vermis
M
N
O
P
P0 Hemisphere
E
Vermis EGL
F
Vermis Rest
CC3 positive cells/mm2
G
Hemisphere EGL
H
Hemisphere Rest
*
**

Supplemental Figure 5. Early postnatal cerebellar hypoplasia is associated with increased apoptosis of non-EGL progenitors. (A-D) Quantification of phosphohistone 3B (PH3B) positive cells, in the EGL and rest of the cerebellar vermis and hemispheres as determined per mm^2 at stages indicated (n=3 per genotype). No significant difference in proliferation was noted in the vermis or hemispheres at any stage. **(E-H)** Quantification of cleaved caspase 3 (CC3) positive cells, in the EGL and rest of the cerebellar vermis and hemispheres per mm^2 at stages indicated (n=3 per genotype). Note the significant increase in apoptosis at postnatal day 0 (P0) in all but the EGL of *Prdm13* deficient mice (F,H). **(I-P)** Examples of CC3 immunostains, counterstained with haematoxylin, to visualise apoptotic cells on sagittal sections through the cerebellar vermis (I,J) and hemispheres (M,N) at P0, anterior to the left. Magnified views of CC3+ cells in the non- EGL cerebellum at P0 indicated by black boxes in corresponding low power views (K,L,O,P). Note the increase in the number of cells undergoing apoptosis in *Prdm13* deficient cerebella (L,P) *P<0.05, **P<0.01, Student's *t* test, Scale bar = 300µm (I, K).

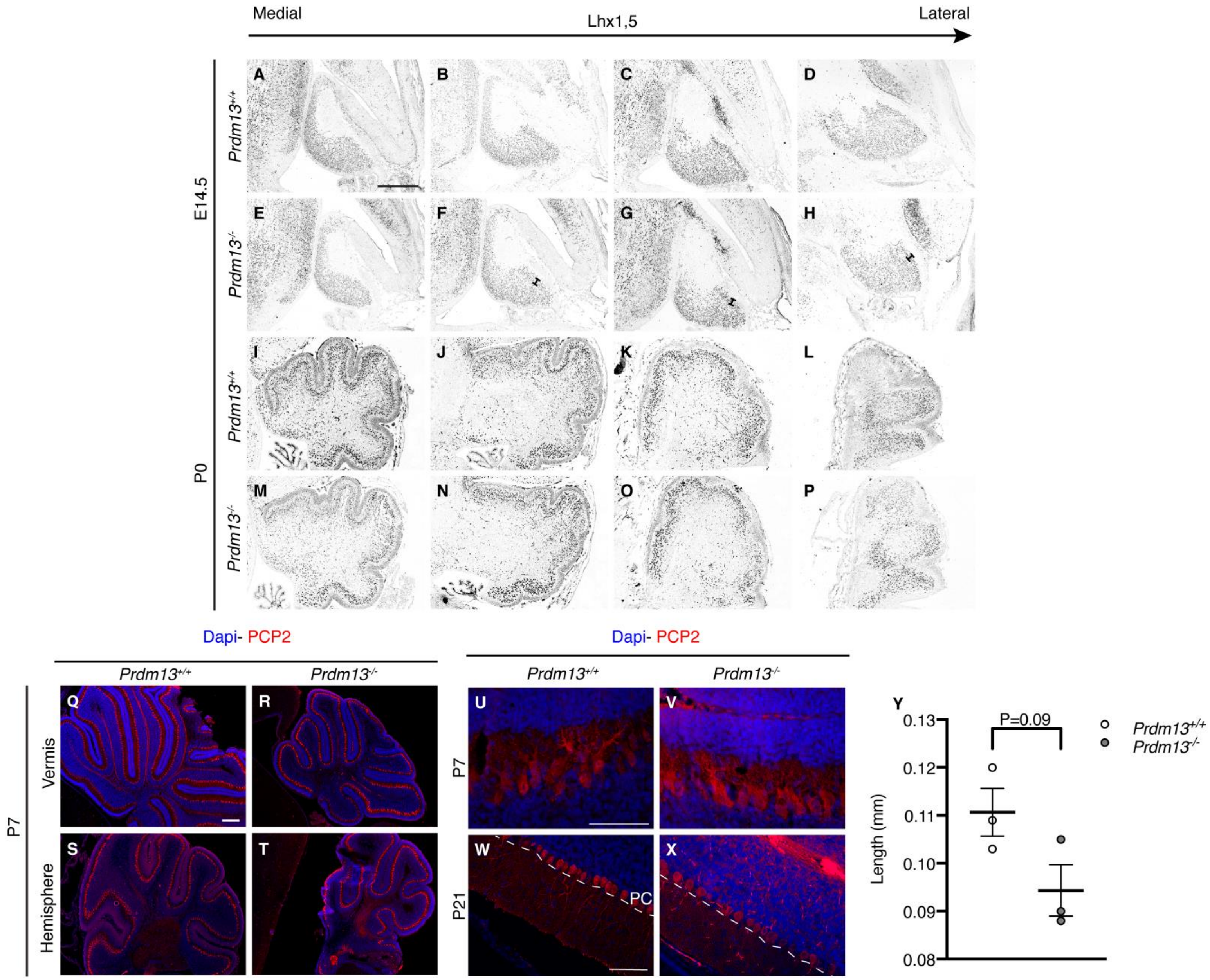

Medial
Lhx1,5
Lateral
E14.5
Prdm13+/+
Prdm13-/-
P0
A B C D
E F G H
I J K L
M N O P
Dapi- PCP2
Prdm13+/+
Prdm13-/-
P7
Vermis
Hemisphere
Q R S T
U V W X
P21
PC
Y
P=0.09
Length (mm)
0.13
0.12
0.11
0.10
0.09
0.08

Supplemental Figure 6. *Prdm13* is not required for PC development. LHX1,5 immunohistochemistry on sagittal sections through the cerebellar vermis and hemispheres at E14.5 (A-H) and P0 (I-P) to label PC progenitors. (Q-T) PCP2 immunochemistry on sagittal sections through the cerebellar vermis and hemispheres at P7 and P21. Note normal PC plate formation in *Prdm13*$^{-/-}$ (F-H) cerebella at E14.5, indicated by brackets. Note normal PC distribution at P0 *(*M-P), normal PC monolayer formation at P7 (R,T) and dendritic arbor at P21 (U-X). (Y) Molecular layer width taken from lobule III/IV to estimate the span of the PC dendritic arbor (n=3 per genotype). Scale bar = 300µm (A, Q), 100µm (U,W). PC = Purkinje cell.

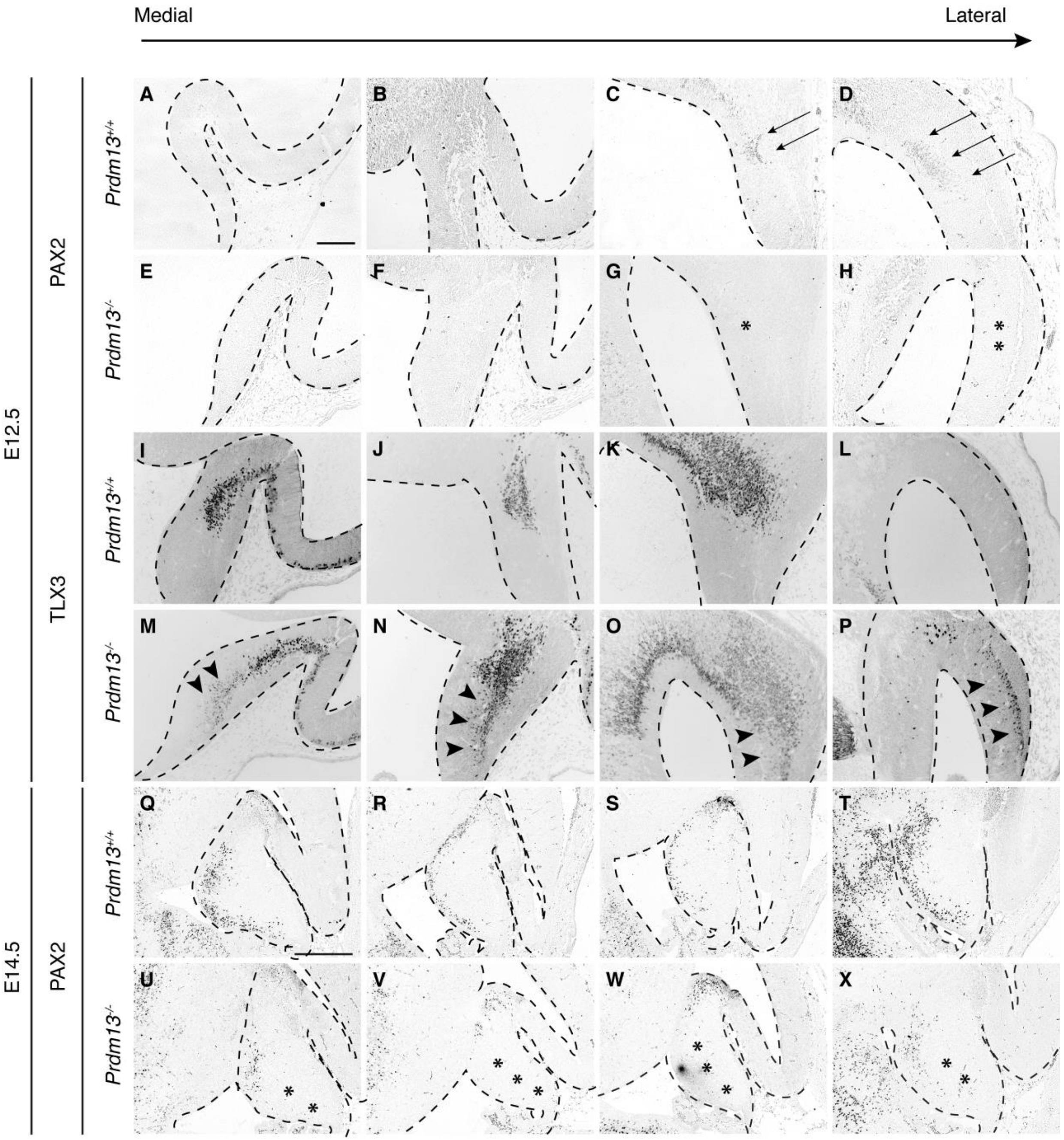

Medial
Lateral
E12.5
PAX2
TLX3
E14.5
PAX2
Prdm13+/+
Prdm13-/-
Prdm13+/+
Prdm13-/-
Prdm13+/+
Prdm13-/-
A
B
C
D
E
F
G
H
I
J
K
L
M
N
O
P
Q
R
S
T
U
V
W
X

Supplemental Figure 7. *Prdm13* is required for early GABAergic fate specification. PAX2 and TLX3 immunostains on sagittal sections through the cerebellum at stages indicated to label GABAergic interneurons and glutamatergic neurons, respectively. (A-P) Note the small cluster of PAX2+ cells in the lateral cerebellum of *Prdm13*$^{+/+}$ mice at E12.5, indicated by arrows (C,D), which were absent from *Prdm13*$^{-/-}$ cerebella at the same stage, indicated by asterisks (G,H). Note that at E12.5 there is expansion of the TLX3+ population laterally (P) and dorsally (M-P) where these cells occupy the length of the ventro-dorsal cerebellum in *Prdm13*$^{-/-}$ mice, indicated by arrowheads (M-P). At E14.5 there is a reduction of PAX2+ neurons in *Prdm13*$^{-/-}$ cerebellar vermis, indicated by asterisks (U-X). Scale bar = 300µm (A, Q).

Supplemental Acknowledgments

We would like to thank GOSgene for organising the exome sequencing and for analysing the subsequent data on Pedigree 1. GOSgene members are as follows: Hywel J. Williams, Polona Le Quesne-Stabej, Louise A. Ocaka, Lamia Boukhibar, Jasmine Gratton, Andrey Gagunashvili, Rojeen Niazi, Georg Otto, Daniel Kelberman, Rosalind Davies, Philip Beales, Chiara Bacchelli, Chela James and Nital Jani. Please note that only the names that are also listed as co-authors in this manuscript directly worked on this project.
